# Single‐Nucleus RNA Sequencing and Spatial Transcriptomics Reveal the Immunological Microenvironment of Cervical Squamous Cell Carcinoma

**DOI:** 10.1002/advs.202203040

**Published:** 2022-08-19

**Authors:** Zhihua Ou, Shitong Lin, Jiaying Qiu, Wencheng Ding, Peidi Ren, Dongsheng Chen, Jiaxuan Wang, Yihan Tong, Di Wu, Ao Chen, Yuan Deng, Mengnan Cheng, Ting Peng, Haorong Lu, Huanming Yang, Jian Wang, Xin Jin, Ding Ma, Xun Xu, Yanzhou Wang, Junhua Li, Peng Wu

**Affiliations:** ^1^ BGI‐Zhenzhen Shenzhen 518083 China; ^2^ Shenzhen Key Laboratory of Unknown Pathogen Identification BGI‐Shenzhen Shenzhen 518083 China; ^3^ Cancer Biology Research Center (Key Laboratory of the Ministry of Education) Tongji Hospital Tongji Medical College Huazhong University of Science and Technology Wuhan 430000 China; ^4^ Department of Gynecologic Oncology Tongji Hospital Tongji Medical College Huazhong University of Science and Technology Wuhan 430000 China; ^5^ College of Life Sciences University of Chinese Academy of Sciences Beijing 100049 China; ^6^ College of Innovation and Experiment Northwest A&F University Yangling 712100 China; ^7^ School of Basic Medicine Qingdao University Qingdao 266071 China; ^8^ Department of Biology University of Copenhagen Copenhagen DK‐2200 Denmark; ^9^ Department of Obstetrics and Gynecology Southwest Hospital Third Military Medical University Chongqing 400038 China; ^10^ China National GeneBank BGI‐Shenzhen Shenzhen 518120 China; ^11^ Guangdong Provincial Key Laboratory of Genome Read and Write Shenzhen 518120 China; ^12^ James D. Watson Institute of Genome Sciences Hangzhou 310058 China

**Keywords:** cancer‐associated fibroblasts, cervical cancer, single‐nucleus RNA sequencing, spatial transcriptomics, tumor microenvironment

## Abstract

The effective treatment of advanced cervical cancer remains challenging. Herein, single‐nucleus RNA sequencing (snRNA‐seq) and SpaTial enhanced resolution omics‐sequencing (Stereo‐seq) are used to investigate the immunological microenvironment of cervical squamous cell carcinoma (CSCC). The expression levels of most immune suppressive genes in the tumor and inflammation areas of CSCC are not significantly higher than those in the non‐cancer samples, except for *LGALS9* and *IDO1*. Stronger signals of CD56^+^ NK cells and immature dendritic cells are found in the hypermetabolic tumor areas, whereas more eosinophils, immature B cells, and Treg cells are found in the hypometabolic tumor areas. Moreover, a cluster of pro‐tumorigenic cancer‐associated myofibroblasts (myCAFs) are identified. The myCAFs may support the growth and metastasis of tumors by inhibiting lymphocyte infiltration and remodeling of the tumor extracellular matrix. Furthermore, these myCAFs are associated with poorer survival probability in patients with CSCC, predict resistance to immunotherapy, and might be present in a small fraction (< 30%) of patients with advanced cancer. Immunohistochemistry and multiplex immunofluorescence staining are conducted to validate the spatial distribution and potential function of myCAFs. Collectively, these findings enhance the understanding of the immunological microenvironment of CSCC and shed light on the treatment of advanced CSCC.

## Graphical abstract

The high heterogeneity in viral gene expression, immune response, and metabolism in cervical squamous cell carcinoma indicates that therapies targeting multiple biological processes may yield better curative effects. The discovery of pro‐tumorigenic cancer‐associated myofibroblasts (myCAFs) in clinical samples suggests that interventions on myCAFs might complement the current treatments for invasive cervical cancer.

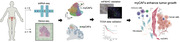

## 1 Introduction

Cervical cancer is the fourth most common cancer affecting women's health globally, especially in low‐income and middle‐income regions.^[^ ^1^, ^2^ ^]^ Approximately 70–80% of cervical cancers are squamous cell carcinoma, 20–25% are adenocarcinomas, and the rest are adenosquamous cell carcinomas and rare histological types, such as mesenchymal carcinoma, neuroendocrine carcinoma, glassy cell carcinoma, and undifferentiated carcinoma.^[^ ^3^, ^4^, ^5^, ^6^ ^]^ The proportion of pathological types of cervical cancer may vary depending on the popularization of human papillomavirus (HPV) vaccines and early screening strategies in specific regions.^[^ ^2^ ^]^ Currently, over 12 types of HPVs are known to be carcinogenic.^[^ ^7^ ^]^ Among them, HPV16 is responsible for 60–70% of cervical cancer cases, especially cervical squamous cell carcinoma (CSCC). Since 2018, the World Health Organization (WHO) has called for the global elimination of cervical cancer, quantifying actions in vaccination, screening, and disease treatment/management,^[^ ^8^ ^]^ which requires joint efforts from different parties for decades.

Although patients with primary cervical cancer undergoing radical hysterectomy can achieve a favorable prognosis, the 5‐year overall survival and disease‐free survival rates associated with advanced cervical cancer are unsatisfactory.^[^ ^9^, ^10^ ^]^ At present, chemotherapy (e.g., paclitaxel, cisplatin, and bevacizumab) and radiotherapy remain the main palliative treatments for patients with metastatic or recurrent cervical cancer, and are associated with a low response rate (48%) and a short survival period (17 months).^[^ ^11^, ^12^, ^13^, ^14^ ^]^ Immunotherapy brings new hope to treating incurable cervical cancer by reversing the exhausted or suppressed immune activities. Immune checkpoint blockade (ICB) drugs targeting programmed cell death 1 (PD1), programmed cell death ligand 1 (PD‐L1), and cytotoxic T lymphocyte antigen 4 (CTLA4) are currently being tested for the treatment of recurrent/metastatic cervical cancers.^[^ ^15^, ^16^, ^17^ ^]^ Unfortunately, the overall response rates to ICB therapy are low, varying from 4% to 26%.^[^ ^15^, ^18^, ^19^, ^20^ ^]^ Clarifying the immune landscape of CSCC, especially the immunosuppression status in the tumor microenvironment (TME), may help us better address this phenomenon and adjust our treatment strategy for cervical cancers.

Among the cell types constituting the TME, cancer‐associated fibroblasts (CAFs) are a major contributor to phenotypic heterogeneity. Depending on their subtypes, these cells can be derived from activated normal fibroblasts, epithelial‐to‐mesenchymal transition (EMT), and endothelial‐to‐mesenchymal transition.^[^ ^21^ ^]^ CAFs can enhance the proliferation, invasion, and metastasis of tumors, as revealed by investigations of pancreatic cancer, cholangiocarcinoma, lung cancer, breast cancer, and other types of cancers.^[^ ^22^, ^23^, ^24^, ^25^, ^26^ ^]^ CAFs can be identified by marker genes such as *ACTA2* (actin alpha 2, smooth muscle; also known as *α*‐SMA), *FAP* (fibroblast activation protein alpha), *PDGFRB* (platelet‐derived growth factor receptor beta), and *S100A4* (S100 calcium‐binding protein A4).^[^ ^27^ ^]^ However, gene expression patterns of CAFs can vary in different subtypes and tumor types. It is also postulated that different subgroups of CAFs may function differently in the TME, for example, one subgroup may be pro‐tumorigenic while other subgroups may be anti‐tumorigenic,^[^ ^28^, ^29^, ^30^ ^]^ which further complicates the TME. The characteristics of CAFs in clinical samples of CSCC await further exploration.

Single‐cell sequencing and spatial transcriptomics are state‐of‐the‐art tools to unravel the cell heterogeneity and microenvironment of tumors; however, applications of such techniques to clinical samples of CSCC remain limited. Hua et al. investigated the intratumoral heterogeneity of CSCC based on tumor tissues and adjacent normal tissues from one patient using single‐cell RNA sequencing (scRNA‐seq) in 2021.^[^ ^31^ ^]^ While there are some comparative studies on changes in the TME before and after chemotherapy, the associated reports are brief without a detailed presentation of the scRNA‐seq data.^[^ ^32^ ^]^ While more transcripts may be generated from scRNA‐seq than from single‐nucleus RNA sequencing (snRNA‐seq), snRNA‐seq data also contain abundant information for cellular diversity investigation,^[^ ^33^ ^]^ and sometimes may yield more cell types due to less dissociation bias.^[^ ^34^ ^]^ At present, atlas studies using snRNA‐seq are well accepted by the scientific community.^[^ ^35^, ^36^, ^37^ ^]^ Therefore, snRNA‐seq was performed with fresh frozen samples in this study. We collected five and eighteen cervical samples for snRNA‐seq and SpaTial Enhanced REsolution Omics‐sequencing (Stereo‐seq), respectively, to investigate the immunological profiles of CSCC.^[^ ^38^ ^]^ Deciphering the immunological microenvironment of CSCC would provide new insights into the treatment of advanced CSCC, which may accelerate cervical cancer elimination.

## 2 Results

### 2.1 snRNA‐seq Data Revealed the Cellular Composition of CSCC

**Figure 1:**
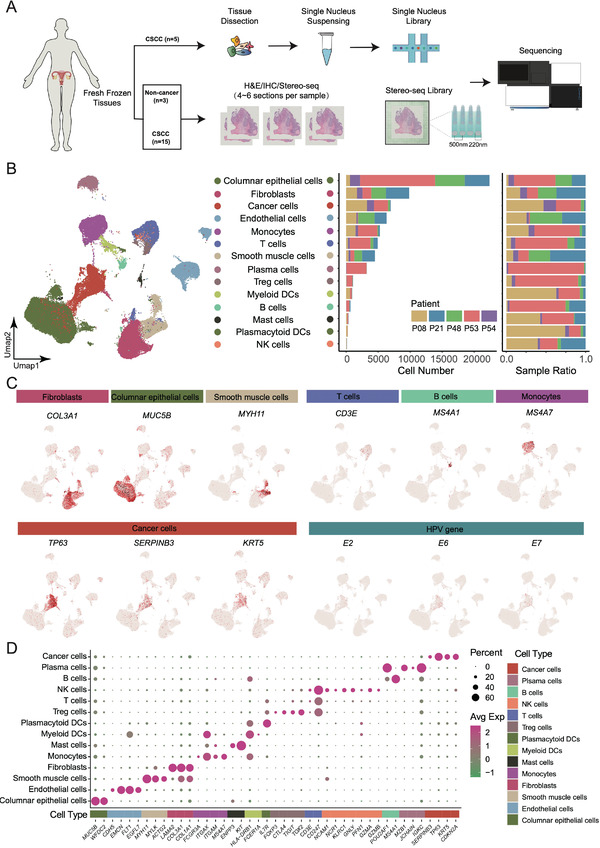
The cellular composition of cervical squamous cell carcinoma (CSCC) tissues. A) Workflow of snRNA‐seq and Stereo‐seq experiments applied to cervical tissues. n indicates the number of samples. B) UMAP of cells identified from the snRNA‐seq data of five CSCC tissues (left). The cell number and proportion of each cell type from each sample (right). C) Expression of selected marker genes and HPV genes in the major cell types of CSCC tissues. D) Expression matrix of cell‐type marker genes in the 14 cell types isolated from CSCC tissues.

To fully characterize the cell composition of the cervical tissues, we collected CSCC samples from five patients for snRNA‐seq (**Figure** **1**A; Figure S1 and Table S1, Supporting Information). A total of 67,003 cells and 30,996 genes passed quality control (Figure 1B; Table S2, Supporting Information), from which we identified 14 cell types based on canonical cell markers (Figure 1B; Table S3, Supporting Information), including cancer cells (6,960), columnar epithelial cells (CECs; 22,396), endothelial cells (6,340), smooth muscle cells (4,502), fibroblasts (9,836), B cells (689), monocytes (5,281), T cells (4,930), regulatory T (Treg) cells (1,081), plasma cells (3,236), myeloid dendritic cells (DCs) (955), plasmacytoid DCs (272), mast cells (384), and natural killer (NK) cells (141). The uterine cervix contains two types of cells lining its surface: stratified squamous epithelial cells on the ectocervix and simple CECs on the endocervix and crypts. Dysplasia of squamous epithelial cells leads to CSCC. Therefore, the cancer cells mainly expressed the known CSCC‐associated gene *SERPINB3* (serpin family B member 3),^[^ ^39^, ^40^ ^]^ tumor genes *TP63*, *CDKN2A*, and keratin gene *KRT5* in squamous cells (Figure 1C,D). The tissues were mainly from patients with advanced cancer (FIGO Stage IB2‐IIIC1), and tissues with no staging information were collected during cervical biopsy before chemotherapy. Therefore, only a few normal epithelial squamous cells were isolated. Mapping of the snRNA‐seq reads against high‐risk HPV reference genomes revealed the presence of viral genes in the cancer cells (Figure 1C, Table S2, Supporting Information). We also identified a large cluster of CECs highly expressing *MUC5B* and *WFDC2*. This cell type is mainly located in the endocervix epithelia but can also appear at the squamocolumnar junction in the adult uterine cervix and some glands. Smooth muscle cells, fibroblasts, and endothelial cells are the major cell types present in the cervical stroma. Smooth muscle cells highly expressed *MYH11*, *MYLK*, *ACTG2*, *COL3A1*, and *COL1A1*, fibroblasts expressed high levels of *LAMA2* in addition to *COL3A1* and *COL1A1*, while endothelial cells could be distinguished by high expression of *EMCN*, *FLT1*, and *EGFLT* (Figure 1C,D). In addition to the structural cells of the cervix, diverse immune cell types were also identified, with monocytes (*ITGAX*, *MX4A7*), T cells (*CD3E*, *CD247*), and plasma cells (*MZB1*, *IGKC*) being the most abundant (Figure 1B,C). In short, the snRNA‐seq data revealed the structural and immune cell composition of the CSCC tissues, facilitating downstream spatial transcriptomic analysis with specific cellular gene expression profiles.

### 2.2 Spatial Transcriptomic Characterization of CSCC

**Figure 2:**
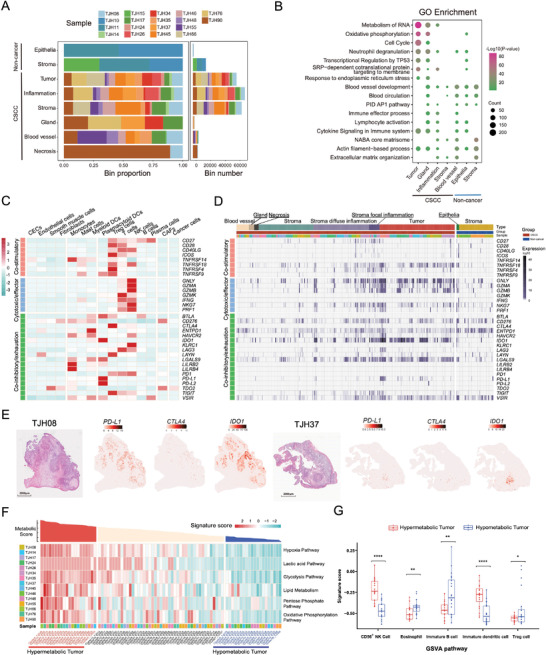
Transcriptomic analysis of the immunity and energy metabolism heterogeneity in cervical squamous cell carcinoma (CSCC). A) Distribution of Stereo‐seq clusters in 18 cervical samples. CSCC (*n* = 15): tumor, stroma, inflammation, gland, blood vessel, and necrosis; non‐cancer (*n* = 3, TJH10, TJH11, and TJH15): epithelia and stroma. B) Gene ontology (GO) enrichment of major Stereo‐seq clusters. C) Heatmap showing the expression of gene sets associated with different immune functions (co‐stimulatory, cytotoxic/effector, and co‐inhibitory/exhaustion) in cell types identified by snRNA‐seq data. D) Heatmap showing the expression of gene sets associated with different immune functions (co‐stimulatory, cytotoxic/effector, and co‐inhibitory/exhaustion) in Stereo‐seq clusters. The stroma areas were labelled according to their lymphocyte infiltration statuses, as assessed by hematoxylin and eosin staining images. Stroma: no obvious lymphocyte infiltration; Stroma diffuse inflammation: diffuse lymphocytes in the stroma; Stroma focal inflammation: aggregation of lymphocytes in the stroma. E) Spatial expression of selected immune checkpoint genes (*PD‐L1*, *CTLA4*, and *IDO1*) in two representative Stereo‐seq samples. The genes tended to be enriched in tumor areas. F) Top, metabolic scores corresponding to the tumor areas of the 15 CSCC samples. Bottom, heatmap showing the GSVA scores of hypoxia, lactic acid, glycolysis, lipid metabolism, pentose phosphate, and oxidative phosphorylation pathways for tumor areas from each Stereo‐seq sample. G) Violin plots showing abundance of CD56^+^ NK cells, eosinophils, immature B cells, immature dendritic cells and Treg cells in hyper‐ (*n* = 10) and hypo‐metabolic (*n* = 10) tumors. The *Y* axis shows the GSVA scores for each cell type. The *p* values were determined by the Wilcoxon signed‐rank test: ns, not significant; *p < 0.05; **p < 0.01; ***p < 0.001; ****p < 0.0001.

Spatial information is critical for understanding cell–cell interactions in tissues, which is unfortunately missing from the snRNA‐seq data. Therefore, we utilized Stereo‐seq to acquire in situ gene expression profiles.^[^ ^38^ ^]^ The Stereo‐seq chips contained capture probes with a 25 bp coordinate identity barcode, a 10 bp molecular identity barcode, and a 22 bp polyT tail for mRNA hybridization. Cervical samples from 2 individuals without cancer and 14 patients with CSCC were obtained and embedded in OCT (Table S1, Supporting Information). Serial cryosections of 10 µm thickness were dissected from each OCT block for Stereo‐seq, hematoxylin and eosin (H&E) staining, and immunohistochemical (IHC) staining (Figure S1A and Table S1, Supporting Information). Finally, 18 Stereo‐seq slides were obtained (**Figure** **2**A and Table S4, Supporting Information). These included three slides from the two non‐cancer individuals and 15 slides from the 14 patients with CSCC. Two patients contributed more than one sample. As the samples were from different anatomical sites, they were all included to compensate for the small sample size. The capture spots in the Stereo‐seq chips were 220 nm in diameter, with a center‐to‐center distance of 500 nm between two adjacent spots. The capture spots were grouped into bins to include sufficient genes for accurate clustering. Our preliminary analysis revealed a much higher RNA abundance in tumor areas than in stroma areas. To ensure sufficient genes for annotation, the median number of gene types per bin was required to be > 1,000 for all chips. To balance the expression differences between tumor and stroma, we annotated the CSCC Stereo‐seq slides at bin100 (100 × 100 spots) to fully demonstrate the tissue composition, which would cover an area of ≈ 49.72 × 49.72 µm. The mean number of genes per bin for the CSCC Stereo‐seq slides ranged from 1,767 to 4152 (Table S4, Supporting Information). Because the three Stereo‐seq slides from the non‐cancer patients had lower gene expression intensity than the CSCC slides, they were annotated at bin200 (99.72 × 99.72 µm). Uniform manifold approximation and projection (UMAP) analysis showed that bin clusters of CSCC and non‐cancer tended to dissociate from each other, whereas those of CSCC displayed some convergence (Figure S1B, Supporting Information).

It is worth noting that most spatial transcriptomic techniques capture RNA from permeabilized tissue overlaid on top of a chip in a liquid system, where the lateral diffusion of RNA is unavoidable.^[^ ^38^, ^41^, ^42^ ^]^ This phenomenon is most obvious in cavities within tissues and tissue areas with extremely low RNA abundance, because the capture probes underneath may be far from saturated. Therefore, we combined pathological assessment and marker gene expression patterns (Figure S1C,D, Supporting Information) to ensure that no obvious lateral diffusion occurred in the stereo chips, and this served as a quality control step. Subsequently, the tissue clusters were annotated using gene expression patterns. Six types of tissue clusters were generally identified in our CSCC samples: tumor, stroma (without obvious inflammation), inflammation (stroma with diffuse inflammation or focal inflammation), gland, blood vessel, and necrosis. Tumor, stroma, and inflammation clusters were widely distributed among the Stereo‐seq slides of the CSCC samples, with certain samples containing necrosis, glands, and blood vessels (Figure 2A). Depending on the gene expression profiles, tissue clusters may be further divided into sub‐clusters with number suffixes (Figure S1C, Supporting Information). To facilitate data interpretation, the stromal areas were further divided according to their lymphocyte infiltration status, which included stroma (no obvious lymphocyte infiltration), stroma with diffuse inflammation (diffuse lymphocytes in the stroma), and stroma with focal inflammation (aggregation of lymphocytes in the stroma), as assessed by H&E staining. It should be kept in mind that a spatial cluster herein was virtually a mixture of cells in a defined area but was designated by its major characteristics. For example, tumor clusters were not purely composed of cancer cells but also contained other cell types, although in small numbers, such as immune cells and fibroblasts. The tissue‐specific genes also displayed spatial patterns. Cancerous squamous cell‐associated genes such *KRT5*, *CDKN2A*, and *SERPINB3* were mainly enriched in the Stereo‐seq tumor areas, *IGKC* and *IGLC2* were enriched in inflammatory areas, *VIM* in stromal areas, *ADRA2A* in blood vessels, and *MUC5B* in glands (Figures S1D and S2, Supporting Information). All 14 patients with CSCC who donated the 15 samples were HPV‐positive, with 85.7% (12/14) infected with HPV16, 7.1% (1/14) with HPV33, and 7.1% (1/14) with HPV58. The HPV reads covered 8–100% (≈ 600–7905 bp) of the viral genome and were mainly identified in the tumor areas (Table S4 and Figures S3 and S4, Supporting Information). Spatial visualization demonstrated varied capture signals of viral genes at different tumor sites of the same sample, with E5, E6, E7, and L1 being frequently observed (Figure S4, Supporting Information). In contrast, only marginal HPV reads were identified in non‐CSCC samples (Table S4, Supporting Information). In general, higher transcriptional and translational activities, cell proliferation, oxidative phosphorylation, and immune responses were observed in the Stereo‐seq tumor areas than in other areas (Figure 2B). The initial manual annotations of the Stereo‐seq slides were used to assist in downstream analysis of the TME.

### 2.3 Variable Immune Inhibition in CSCC

To understand the low response rates of cervical cancer to ICB therapy,^[^ ^14^ ^]^ we decided to scrutinize the immune landscape of CSCC for clues. The expression profiles of three gene sets with different immune functions, that is, co‐stimulatory, cytotoxic/effector, and co‐inhibitory/exhaustion, as summarized in the literature review and previous studies^[^ ^43^, ^44^, ^45^ ^]^ were evaluated using both snRNA‐seq (Figure 2C) and Stereo‐seq data (Figure 2D; Table S5, Supporting Information). At the single‐cell level, co‐stimulatory genes were expressed in cells of both innate and adaptive immunity, especially in Tregs, T cells, and NK cells (Figure 2C). Treg cells highly express *CD27, CD28, CD40LG, ICOS, TNFRSF18, TNFRSF4*, and *TNFRSF9*. While these genes are necessary for the maturation and normal suppressive function of Treg cells, the overexpression of *CD27* in Treg cells may restrain the anti‐tumor immune response.^[^ ^46^, ^47^ ^]^ Spatially, co‐stimulatory genes tended to be enriched in the tumor and inflammation areas (Figure 2D). Especially, *TNFRSF18* (also known as glucocorticoid‐induced TNF receptor, *GITR*) was commonly expressed in both inflammatory and tumor areas. However, its expression was also up‐regulated in the epithelia of the non‐cancerous samples. In our snRNA‐seq data, this gene was detected mainly in Tregs, NK cells, T cells, and mast cells. Although *TNFRSF18* is associated with immune suppression by Treg cells in tumors,^[^ ^48^, ^49^ ^]^ its high spatial expression level may be attributed to multiple types of immunocytes. The immune cytotoxic/effector genes were mainly expressed by T and NK cells, some of which were commonly up‐regulated in the inflammation and tumor regions in the Stereo‐seq slides, including *GNLY, GZMA, GZMB*, and *NKG7* (Figure 2D). These genes were mainly expressed by NK cells (Figure 2C), indicating their important roles in the cytotoxic response against CSCC. For the co‐inhibitory/exhaustion genes, we failed to detect any prominent expression of *CTLA4* and *PD‐1* in our Stereo‐seq data (Figure 2D), although *CTLA4* was highly expressed by Treg cells and *PD‐1* by Treg, T, and NK cells (Figure 2C). *PD‐L1*, which is mainly expressed by plasmacytoid DCs, was only overexpressed in the tumor or inflammation areas of a small fraction of Stereo‐seq samples. While *CD276, ENTPD1, IDO1, LGALS9*, and *VSIR* were commonly detected in the Stereo‐seq samples, only *IDO1* and *LGALS9* seemed to have higher and wider expression in the CSCC samples than in the non‐cancer samples (Figure 2D). These two genes were expressed by DCs (Figure 2C) and down‐regulated cytotoxic T cell activity.^[^ ^50^, ^51^ ^]^ Whether *IDO1* and *LGALS9* could be better targets for ICB therapy against CSCC than *CTLA4* and *PD‐L1* remains to be explored. Moreover, when we zoomed in to check the immune genes in the same Stereo‐seq slide, their expression varied greatly between different tumor areas. In sample TJH08, the tumor areas commonly expressed high *IDO1*, low *PD‐L1*, and very low *CTLA4* (Figure 2E). In contrast, only one tumor area in the sample TJH37 expressed these genes (Figure 2E and Figure S2, Supporting Information). Collectively, although both our snRNA‐seq and Stereo‐seq data displayed evidence of immune exhaustion in patients with CSCC, the immune microenvironment varied considerably between and within patients.

### 2.4 Energy Metabolic Statuses of Tumors were Associated with Different Immune Responses

Energy metabolism is one of the hallmarks of cancer and is closely associated with the oxygen status and energy production pathways.^[^ ^52^, ^53^ ^]^ Tumor metabolic behaviors can also modulate the immune microenvironment of tumors, and serve as putative intervention targets for cancer therapy.^[^ ^53^, ^54^ ^]^ Therefore, we selected six associated pathways, including hypoxia, lactic acid, glycolysis, lipid metabolism, pentose phosphate, and oxidative phosphorylation, to explore their spatial activities in our CSCC samples. Gene set variation analysis (GSVA) was conducted separately for the six pathways (Table S6, Supporting Information), and the mean GSVA scores for the six pathways were calculated as the metabolic score for each tumor area. Based on the GSVA metabolic score, the Stereo‐seq tumor clusters ranked among the top 20 were categorized as hypermetabolic tumors, while those ranked among the last 20 were categorized as hypometabolic tumors (Figure 2F). Generally, the hypermetabolic tumors displayed much higher activities in the oxidative phosphorylation, glycolysis, and lactic acid pathways, indicating active aerobic glycolysis in proliferating cancer cells, that is, the Warburg effect. Moreover, the hypermetabolic tumors were accompanied by severe hypoxia and active lipid metabolism, suggesting intense oxidative and nutrient stress in fast‐growing tumors. To further explore the relationship between metabolism and immune response, the GSVA signature scores for different immunocytes in each bin within the hyper‐ and hypometabolic tumor areas were calculated. Based on the average signature score for each tumor area, significant differences were observed for several cell types (Figure 2G). CD56^+^ NK cells and immature DCs showed much stronger signals in the hypermetabolic tumor areas than in the hypometabolic ones, indicating that hypermetabolic tumors might be more prone to be associated with innate immune responses. At the same time, there were more eosinophils, immature B cells, and Treg cells in the hypometabolic tumor areas, suggesting an ineffective adaptive immune response. Two of the Stereo‐seq samples, TJH34 and TJH35, contained both hyper‐ and hypometabolic tumor areas (Figure 2F), indicating high intra‐individual heterogeneity in tumor metabolism.

### 2.5 Characterization of Cancer Associated Myofibroblasts (myCAFs) in CSCC with snRNA‐seq Data

**Figure 3:**
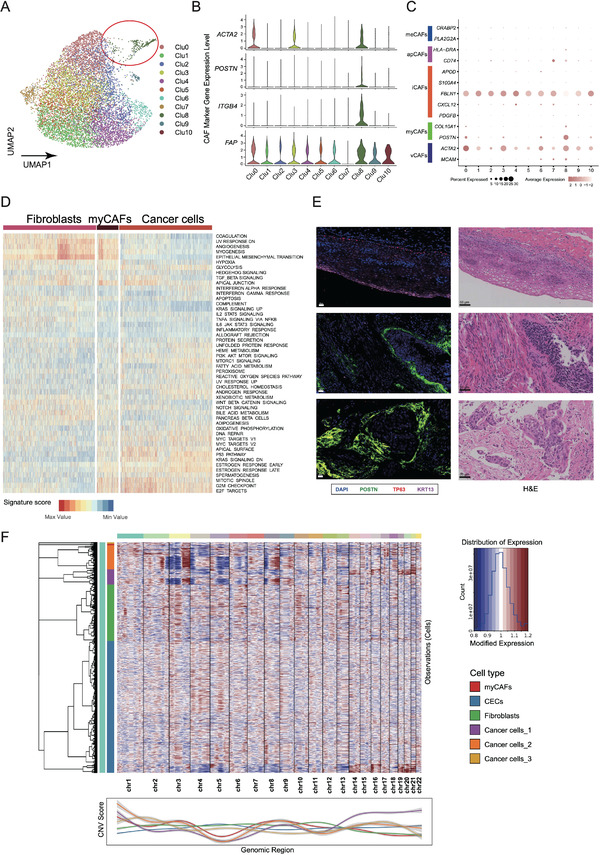
Identification of cancer‐associated myofibroblasts (myCAFs) in cervical squamous cell carcinoma (CSCC). A) UMAP of 9836 fibroblasts. CAFs are outlined in red. B) Violin plot showing the expression levels of *ACTA2*, *POSTN*, *ITGB4*, and *FAP* in the 10 clusters of fibroblasts. C) Dot plot showing the expression levels of marker genes for different subtypes of CAFs in the subclusters of fibroblasts. Abbreviations: meCAFs, CAFs with a highly activated metabolic state; apCAFs, antigen‐presenting CAFs; iCAFs, inflammatory CAFs; myCAFs, cancer associated myofibroblasts; vCAFs, vascular CAFs. D) GSVA results of fibroblasts (9,602 cells), myCAFs (234 cells), and cancer cells (6,960 cells). E) Multiplex immunofluorescence (left panel) and hematoxylin and eosin (right panel) staining of serial FFPE tissue sections showing the presence of myCAFs in CSCC tissues. The differentiated squamous cells (top) were intensely stained by KRT13 (violet); myCAFs were stained by POSTN (green, middle, and bottom). TP63 (p63, red), a key protein controlling epidermal morphogenesis, was positively stained in the nuclei of some basal epithelial cells (top). F) Comparison of copy number variation (CNV) between cancer cells (6,960 cells), myCAFs (234 cells), the other fibroblasts (9,602 cells), and CECs (22,396 cells). The upper panel shows the CNV statuses of each cell from chromosome 1 to 22. The lower panel displays the CNV profiles for subclusters of cancer cells, myCAFs, the other fibroblasts, and CECs, which are shown as a smoothed curve of the mean CNV score of all the cells within each cell group.

When exploring the immune differences between the hyper‐ and hypo‐metabolic tumor areas in sample TJH34, we noticed a unique spatial cluster outside the hypermetabolic tumor regions. This cluster was different from most stromal clusters and looked like a ribbon enclosing the tumor. Because this cluster was part of the stroma, we closely scrutinized the fibroblasts in our snRNA‐seq data. Fortunately, we identified a small set of CAFs (234 cells) derived from all five samples (**Figure** **3**A) that highly expressed *ACTA2*, *POSTN* (periostin, a secreted ECM protein), *ITGB4*, and *FAP* (Figure 3B). While multiple phenotypes of CAFs have been reported, these CAFs mainly expressed marker genes of myofibroblasts and were defined as myCAFs (Figure 3C).^[^ ^55^, ^56^, ^57^, ^58^ ^]^ Functional enrichment based on hallmark gene sets (MSigDB v7.4, https://www.gsea‐msigdb.org/gsea/msigdb/) showed that the myCAFs shared common activities with both fibroblasts and cancer cells (Figure 3D). The myCAFs were involved in pathways similar to those associated with fibroblasts, including UV response down‐regulation, angiogenesis, myogenesis, and EMT. For pathways including the p53 pathway, KRAS signaling down‐regulation, estrogen response, mitotic spindle, G2/M checkpoint, and E2F targets, myCAFs showed similar activities as cancer cells. At the gene level, myCAFs not only highly expressed marker genes for fibroblasts, such as the collagen protein family (*COL1A1, COL3A1, COL4A1, COL5A2*, and *COL6A3*), but also genes for proliferating cells, such as *TOP2A* and *MKI67* (Table S7, Supporting Information). These results indicate that myCAFs may undergo active proliferation to construct the TME. Multiplex immunofluorescence (mIF) staining was used to determine the presence of myCAFs in CSCC tissues. While *ACTA2*, *POSTN*, *ITGB4*, and *FAP* all serve as markers of myCAFs, *POSTN* displayed the highest specificity. *ACTA2* and *FAP* were also expressed in other subtypes of fibroblasts (Figure 3B). Though not highly expressed by the other fibroblasts, spatial data revealed the presence of *ITGB4* in some tumor or stromal regions. Moreover, *POSTN* has been linked to pro‐invasive CAFs in CSCC and other cancers.^[^ ^59^, ^60^, ^61^, ^62^ ^]^ In contrast, the association between *ITGB4* and CSCC progression was less pronounced. Therefore, we chose *POSTN* as a single marker gene for myCAFs rather than the other three genes for staining. The differentiated squamous cells and some tumors were intensely stained by KRT13, whereas the myCAFs were stained by POSTN (Figure 3E). TP63 (p63), a key protein controlling epidermal morphogenesis,^[^ ^63^ ^]^ was positively stained in the nuclei of some basal epithelial cells and cancer cells, depending on the concordance between the protein isoforms and the antibody.^[^ ^64^ ^]^ The staining pattern of POSTN confirmed the presence of myCAFs adjacent to the tumors (Figure 3E, bottom).

**Figure 4:**
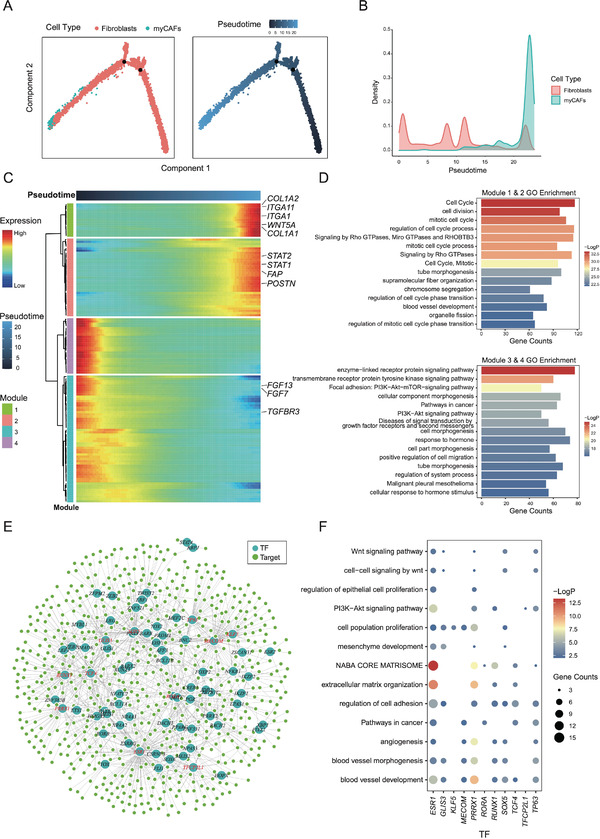
Pseudotime and regulatory network analysis of myCAFs in CSCC. A) Pseudotime trajectory of myCAFs (234 cells) and the other fibroblasts (9,602 cells). B) Density distribution of myCAFs and the other fibroblasts along the pseudotime trajectory. C) DEGs between myCAFs and the other fibroblasts with pseudotime variation. D) GO enrichment of the gene modules with pesudotime variation. E) Regulatory network of myCAFs. Eleven genes in red are regulatory factors (TFs) with over 30 target genes. F) GO enrichment of the target genes of the 11 TFs in myCAFs.

Large‐scale chromosomal copy number variation (CNV) analysis showed that myCAFs shared similarities with both normal cells (Figure 3F, chromosome 14–22) and some cancer cells (chromosome 3–9). Pseudotime analysis on myCAFs and the other fibroblasts showed that myCAFs located at one end of the trajectory (**Figure** **4**A,B).^[^ ^108^ ^]^ A total of 2,543 genes (*p* < 0.05) associated with the pseudotime trajectory of myCAFs were identified (Table S8, Supporting Information), which can be divided into four modules. Figure 4C shows the top 100 genes with pseudotime variation. Genes of Modules 1 and 2 tended to be up‐regulated in myCAFs, such as *COL1A1, COL1A2, ITGA1, ITGA11, FAP*, and *POSTN*, which were components and markers of myCAFs.^[^ ^65^, ^66^ ^]^ Meanwhile, genes promoting tumor growth were also up‐regulated in myCAFs, such as *WNT5A, STAT1*, and *STAT2*.^[^ ^67^, ^68^, ^69^ ^]^ Modules 1 and 2 were mainly associated with cell cycle processes, tube morphogenesis, and blood vessel development (Figure 4D, top). Meanwhile, genes of Modules 3 and 4 displayed a trend of down‐regulation in myCAFs (Figure 4C), such as *FGF7, FGF13*, and *TGFBR3*. These two modules were also associated with multiple signaling pathways linked with cancer, such as PI3K−Akt and PI3K−Akt−mTOR signaling pathway (Figure 4D, bottom).^[^ ^70^ ^]^ Regulatory network analysis of myCAFs with GENIE3 revealed 1,242 regulatory pairs (Table S9, Supporting Information, weight > 0.05).^[^ ^110^ ^]^ Among them, 11 transcription factors (TFs) targeting more than 30 genes (Figure 4E), which included *ESR1, GLIS3, KLF5, MECOM, PRRX1, RORA, RUNX1, SOX5, TCF4, TFCP2L1*,and *TP63*. These TFs not only participated in the regulation of extracellular matrix and cell adhesion but were also engaged in critical pathways in tumorigenesis such as angiogenesis, blood vessel development, blood vessel morphogenesis, epithelial cell proliferation, PI3K−Akt signaling pathway, and Wnt signaling pathway (Figure 4F).^[^ ^71^, ^72^ ^]^ These characteristics indicate a critical role of myCAFs in the development of CSCC.

### 2.6 The myCAFs Might Facilitate the Growth and Metastasis of CSCC from Diverse Aspects

**Figure 5:**
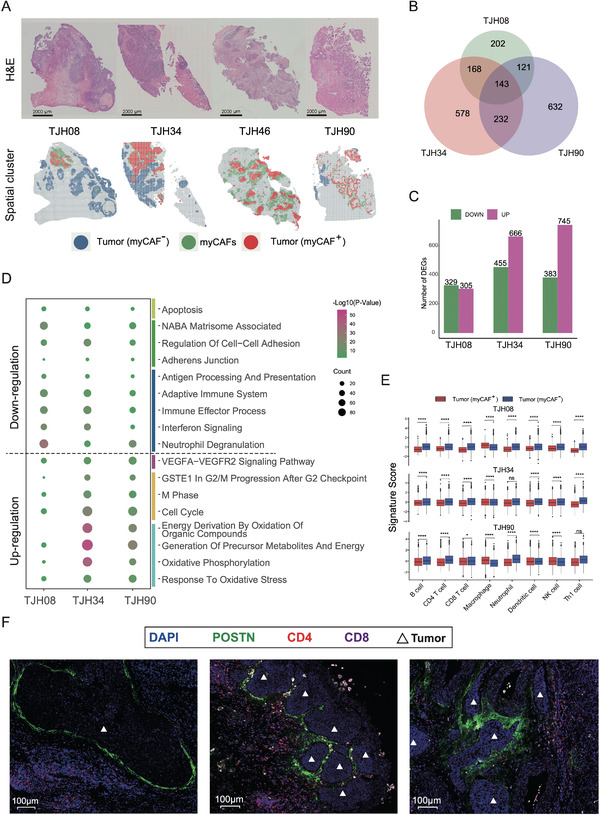
Spatial and functional characterization of myCAFs in CSCC. A) Spatially projected myCAFs in representative Stereo‐seq slides. The projected area was determined based on the MIA score of myCAFs, POSTN expression pattern, and the IHC staining results of POSTN (see Figure S5B,C, Supporting Information). B) Venn map showing the number of DEGs identified in the three Stereo‐seq samples with both myCAF^+^ and myCAF^–^ tumors. C) Bar plot showing the numbers of up‐ and down‐regulated genes in three Stereo‐seq samples. The myCAF^+^ tumors were compared to the myCAF^–^ tumors. D) Dot plot of enriched GO terms for up‐ and down‐regulated DEGs identified in (C). E) Box plot showing the abundance of immune cells in myCAF^+^ and myCAF^–^ tumors in three samples. TJH08, 5428 bins (472 myCAF^+^ tumor bins and 4956 myCAF^–^ tumor bins); TJH34, 4617 bins (1905 myCAF^+^ tumor bins and 2712 myCAF^–^ tumor bins); TJH90, 3863 bins (2729 myCAF^+^ tumor bins and 1134 myCAF^–^ tumor bins). The *p* values were determined by Student's *t* test: ns, not significant; *p < 0.05; **p < 0.01; ***p < 0.001; ****p < 0.0001. F) mIF staining showing the aggregation of CD4^+^ and CD8^+^ immune cells in the stroma adjacent to tumors enclosed by myCAFs. Tumors can be recognized by the densely packed nuclei stained by DAPI (blue), indicated by white triangles. Immune cells were stained by CD4 (red) and CD8 (violet). myCAFs were stained by POSTN (green).

To locate the spatial distribution of myCAFs in Stereo‐seq chips, we adopted the multimodal intersection analysis (MIA) approach developed by Moncada et al. to integrate snRNA‐seq and Stereo‐seq data.^[^ ^111^ ^]^ Briefly, this method calculated the overlapping degree of the expression levels of cell type‐specific genes identified by snRNA‐seq data and the area‐specific genes characterized by Stereo‐seq data. The smaller the resultant *p*‐value, which is referred to as the MIA score in our later description, the stronger the correlation between a defined cell type and a spatial area. The initial MIA results showed that our Stereo‐seq clustering results complied with the expected cell composition in the corresponding areas (Figure S5A, Supporting Information). Unfortunately, the MIA score alone cannot fully reflect the spatial specificity of the cells, especially in areas with low RNA abundance. Therefore, a high expression level of *POSTN* and a high MIA score for myCAFs were simultaneously utilized to define Stereo‐seq clusters of myCAFs (Figure S5B, Supporting Information). The results showed that myCAFs were enriched around some tumor areas in 4 out of the 15 Stereo‐seq slides (**Figure** **5**A). The presence of myCAFs in CSCC was further confirmed by IHC staining of POSTN using serial tissue sections from the same samples (Figure S5C, Supporting Information). Notably, not all tumor areas were surrounded by myCAFs, making us curious about the biological differences associated with the presence of these cells.

To comprehensively reveal the biological functions of myCAFs in CSCC, we divided the tumor areas in the Stereo‐seq slides into two types: tumor areas surrounded by myCAFs (myCAF^+^ tumors) and tumor areas not surrounded by myCAFs (myCAF^–^ tumors). The existence of myCAFs was jointly determined by the MIA approach and *POSTN* expression pattern, as previously mentioned. Three Stereo‐seq slides were found to contain both myCAF^+^ and myCAF^–^ tumor areas and were used for downstream analysis. We then used the differentially expressed genes (DEGs) between the myCAF^+^ and myCAF^–^ tumor areas (|Log_2_FC| > 0.25, *p <* 0.05) of the three samples to perform GO enrichment analysis (Figure 5B,C; Table S10, Supporting Information). The results showed that the myCAF^+^ tumors were more active in energy usage, metabolism, mitosis, and cell growth than myCAF^–^ tumors (Figure 5D). Meanwhile, cellular adhesion, apoptosis, and immune responses were down‐regulated in myCAF^+^ tumors. These observations coincided with the immune and metabolic heterogeneity of CSCC (Figure 2F,G). These results indicate that the presence of myCAFs may support tumor progression from different aspects.

Next, we calculated the gene module expression scores of immune gene sets for the tumor bins to evaluate the immune cell abundance. The results showed significantly reduced numbers of B cells, CD4^+^ T cells, CD8^+^ T cells, neutrophils, DCs, NK cells, and Th1 cells in myCAF^+^ tumors (Figure 5E and Figure S5D, Supporting Information), indicating that myCAFs might act as a physical barrier to prevent the infiltration of pro‐immunity cells into tumor areas. To confirm this, we conducted mIF to measure the spatial distribution of myCAFs, CD4^+^ cells, and CD8^+^ cells using POSTN, CD4, and CD8 as markers, respectively. The results showed the accumulation of CD4^+^ and CD8^+^ cells outside the tumors enclosed by myCAFs, especially CD4^+^ cells (Figure 5F). While more tumor‐associated macrophages (TAM) were identified in myCAF^+^ tumor areas (Figure 5E), the distribution of M1 (tumor‐suppressive) and M2 (tumor‐promoting) phenotypes showed the opposite trend (Figure S5E, Supporting Information).^[^ ^73^ ^]^ Due to the small sample size and weak signal of macrophages, we were unsure of the relationship between myCAFs and TAMs of different phenotypes.

**Figure 6:**
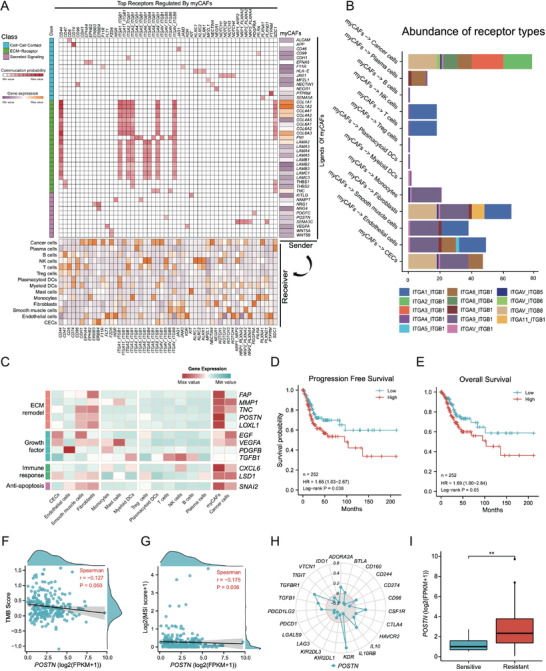
Functional analysis of myCAFs in CSCC. A) Ligand‐receptor communication network between myCAFs and different cervical cells predicted by snRNA‐seq data. The cell–cell communication probability was estimated by integrating gene expression with prior knowledge of the interactions between signaling ligands, receptors and their cofactors. Right, heatmap of the top predicted ligands expressed by myCAFs. Middle, heatmap of ligand‐receptor pairs between myCAFs and different cell types in cervical squamous cell carcinoma (CSCC). Bottom, expression heatmap of top receptors regulated by myCAFs in different cell types. CECs, columnar epithelial cells; ECM, extracellular matrix. B) Bar plot showing the integrin types involved in potential communications between myCAFs and the other cell types. C) Expression heatmap of gene sets related to functions of myCAFs in tumor development in snRNA‐seq data. D) The progression‐free and overall E) survival probabilities of patients with CSCC estimated based on the signature scores of the marker gene set for myCAFs. The analysis was conducted using a 252 CSCC dataset from TCGA. F) The relationships between POSTN expression and TMB scores in the TCGA dataset (*n* = 252). G) The relationships between POSTN expression and MSI scores in the TCGA dataset (*n* = 252). H) The relationships between POSTN expression and common immune genes associated with immunotherapy in the TCGA dataset (*n* = 252). I) Differential expression of *POSTN* in immunotherapy sensitive (*n* = 10) and resistant (*n* = 242) groups. Expression data presented as mean ± SEM, *p* values were calculated using the Wilcoxon rank sum test: ns, not significant; *p < 0.05; **p < 0.01; ***p < 0.001; ****p < 0.0001.

As part of the stroma, myCAFs must interact closely with cancer, stromal, and immune cells. Indeed, analysis of the snRNA‐seq data showed potential interactions between myCAFs and other cells regarding extracellular matrix (ECM) formation and cell–cell contact (**Figure** **6**A). The myCAFs highly expressed genes of the collagen family, especially *COL1A1, COL1A2, COL4A1, COL4A2, COL4A5, COL6A1, COL6A2*, and *COL6A3*, which might interact with CD44 expressed by immunocytes and smooth muscle cells to modulate cell adhesion and migration. Collagens may also interact with diverse members of the integrin family expressed by cancer, immune, and stromal cells. Similarly, FN1 (fibronectin 1, a soluble glycoprotein) and laminins (*LAMA2, LAMA3, LAMA4, LAMA5, LAMB1, LAMB2, LAMB3, LAMC1*, and *LAMC3*) expressed by myCAFs might also interact with other cell types through integrins. Integrins are membrane receptor proteins composed of *α* and *β* subunits that are involved in cell adhesion and recognition. The myCAFs seem to use different heterodimeric forms of integrins to interact with other cell types. They may interact with cancer cells through integrins composed of subunits *α*2*β*1, *α*3*β*1, and *α*v*β*8, while interacting with endothelial cells, CECs, and smooth muscle cells through integrins composed of subunits *α*9*β*1, *α*6*β*1, and *α*1*β*1, and with T cells and NK cells through integrins composed of subunits *α*1*β*1 (Figure 6B). Importantly, myCAFs might take advantage of F11R (also called JAM1, junctional adhesion molecule 1) to form tight junctions with cancer and stromal cells through F11R and JAM3, which might prevent the infiltration of immunocytes. myCAFs may also express other matrix proteins including THBS1 (thrombospondin 1), THBS2, and TNC (tenascin C, a matrix protein), to communicate with immunocytes, smooth muscle cells, cancer cells, and plasma cells through CD44, integrin (*α*3*β*1), and SDC1. Moreover, myCAFs overexpressed several tissue‐remodeling factors (Figure 6C), including POSTN, FAP (fibroblast activation protein, a serine protease), MMP1 (matrix metalloproteinase 1), TNC, and LOXL1 (lysyl oxidase like 1, catalyzes the cross‐linking of collagen and elastin). This evidence suggests that myCAFs play a critical role in shaping the tumor extracellular environment.

In addition to their role in ECM construction, myCAFs may also enhance the stemness and proliferation of cancer cells by overexpressing secreted factors, including *SEMA3C*, *POSTN*, and *CXCL6* (Figure 6A,C). *SEMA3C* promotes cancer stem cell maintenance, angiogenesis, and invasion.^[^ ^74^, ^75^ ^]^ *POSTN* augments cancer cell survival by activating the Akt/PKB pathway through integrin *α*v*β*3.^[^ ^62^ ^]^ It may also promote cancer growth through the PTK7‐Wnt/*β*‐catenin signaling pathway.^[^ ^61^ ^]^ *CXCL6* (C‐X‐C motif chemokine ligand 6), which is mainly related to the immune response, has been reported to promote the growth and metastasis of esophageal squamous cell carcinoma (Figure 6C).^[^ ^76^ ^]^ Another highly expressed gene in myCAFs, *SNAI2* (Slug), a snail‐related zinc finger transcription factor, may inhibit apoptosis and promote cancer progression.^[^ ^77^, ^78^ ^]^ Several common growth factors such as transforming growth factor beta 1 (*TGFB1*), epidermal growth factor (*EGF*), and vascular endothelial growth factor A (*VEGFA*) were also expressed by myCAFs (Figure 6C). Moreover, the upregulation of histone lysine demethylase 1 (*LSD1*) in myCAFs might inhibit IFN activation to evade immune attack.^[^ ^79^ ^]^ The Wnt5a signaling protein produced by myCAFs might also suppress the immune response to facilitate tumor metastasis (Figure 6A).^[^ ^80^, ^81^ ^]^ In summary, the myCAFs might be able to potentiate the TME and promote tumor progression.

### 2.7 The Presence of myCAFs was Associated with Poorer Clinical Status of CSCC

To verify the pro‐tumorigenic effects of myCAFs, we first performed survival analyses using a dataset from The Cancer Genome Atlas (TCGA), which contained 252 patients with CSCC.^[^ ^82^ ^]^ The GSVA score for myCAFs was calculated for each patient with CSCC using the marker gene set (*ACTA2*, *POSTN*, *ITGB4*, and *FAP*). Not surprisingly, higher myCAF signals predicted unfavorable progression‐free survival (Figure 6D, HR = 1.66, 95%CI = 1.03–2.67, *p =* 0.038) and overall survival (Figure 6E, HR = 1.69, 95%CI = 1.00‐2.84, *p =* 0.05) for patients with CSCC. Interestingly, survival analysis using *POSTN* alone showed that its high expression was significantly associated with poorer survival (Figure S6A–D, Supporting Information), whereas the expression level of *ITGB4* failed to yield a significant difference in the survival of patients with CSCC (Figure S6E–H, Supporting Information), suggesting that *POSTN* could serve as a reliable molecular and functional indicator of myCAFs.

Currently, there are three commonly used indicators for predicting the effectiveness of immunotherapy, that is, tumor mutation burden (TMB), microsatellite instability (MSI), and immune inhibitory genes (e.g., *PD1/PD‐L1, CTLA‐4*). Patients with a higher level of TMB, MSI, or immune inhibitory gene expression tended to be more sensitive to immunotherapy.^[^ ^83^, ^84^, ^85^ ^]^ Herein, we explored the relationships between myCAFs and the resistance to immunotherapy in patients with CSCC from the TCGA database. The expression levels of *POSTN* were negatively correlated with the TMB (Figure 6F, *p* = 0.05) and MSI (Figure 6G, *p* = 0.006) scores in patients with CSCC. The expression of *POSTN* was also negatively correlated with canonical immune inhibitory genes such as *PDCD1, TIGIT, IDO1*, and *LAG3* (Figure 6H). Intriguingly, the expression of *POSTN* was positively associated with *KDR* (also known as *VEGFR*, vascular endothelial growth factor receptor), which may enhance angiogenesis and induce immunosuppression during cancer metastasis.^[^ ^86^ ^]^ We further used ImmuCellAI to predict the response to immunotherapy of 252 patients with CSCC from the TCGA database.^[^ ^116^ ^]^ Only ten patients were predicted to be sensitive to immunotherapy, whereas the remaining 242 were resistant. The expression of *POSTN* in the sensitive group was significantly lower than in the resistant group (Figure 6I). Collectively, the myCAFs may curtail the sensitivity of patients with CSCC to ICB, and angiogenesis inhibitors might help strengthen the effects of immunotherapy.

**Figure 7:**
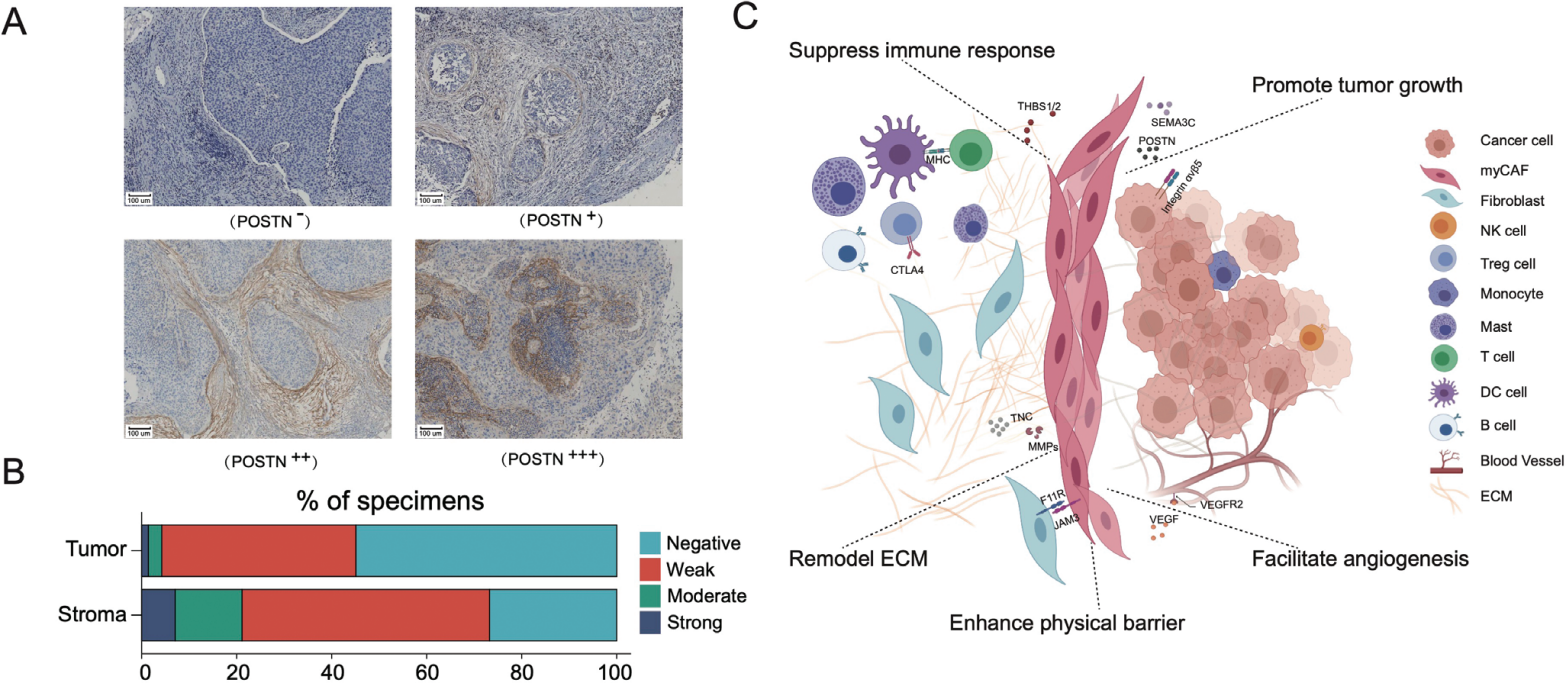
Clinical prevalence and function of myCAFs in CSCC. A) Representative immunohistochemical (IHC) staining patterns of POSTN in the stroma adjacent to tumor areas in FFPE CSCC samples. B) Bar plot showing the IHC staining intensities of POSTN within the tumor area or in the stroma area around tumors in 71 FFPE CSCC samples. C) Schematic summary of the pro‐tumorigenic functions of myCAFs in CSCC. The plot was created using BioRender (https://biorender.com/).

We further measured the protein expression levels of POSTN in the stromal and tumor regions using an independent sample set consisting of 71 archived formalin‐fixed paraffin‐embedded (FFPE) CSCC samples (**Figure** **7**A). We assigned POSTN staining scores to each sample based on the staining intensity and distribution pattern. Although the overall positive rate of POSTN expression (weak, moderate, and strong) in the stroma was much higher than that in the tumor regions, only 21.1% (15/71) of the samples showed moderate/strong staining of POSTN in the stroma around the tumor (Figure 7B). The chi‐square test showed that higher POSTN expression levels in the stroma were significantly correlated with more advanced pathological stages, poorer differentiation, larger tumor size, higher squamous cell carcinoma antigen concentrations in the peripheral blood, and older age (**Table** **1**), which further confirmed the pro‐tumorigenic ability of myCAFs.

**Table 1:** Stromal POSTN expression characteristics in 71 FFPE CSCC samples

Characteristic	IHC score ≥ 4	IHC score < 4	*p*
Number of patients	27 (38.03%)	44 (61.97%)	
Pathologic stage, *n* (%)			0.012
I	5 (17.2%)	24 (82.8%)	
II	10 (50%)	10 (50%)	
III	9 (56.2%)	7 (43.8%)	
NA	3 (50%)	3 (50%)	
Lymph node metastasis, *n* (%)			0.270
≥N1	10 (50%)	10 (50%)	
N0	13 (31.7%)	28 (68.3%)	
NA	4 (40%)	6 (60%)	
Differentiation, *n* (%)			0.007
Low	16 (57.1%)	12 (42.9%)	
Well/moderate	8 (21.6%)	29 (78.4%)	
NA	3 (50%)	3 (50%)	
Tumor size, *n* (%)			0.040
<4 cm	10 (30.3%)	23 (69.7%)	
≥4 cm	10 (66.7%)	5 (33.3%)	
NA	7 (30.4%)	16 (69.6%)	
SCC, *n* (%)			0.030
≤1.5 mg mL^−1^	3 (15%)	17 (85%)	
>1.5 mg mL^−1^	16 (48.5%)	17 (51.5%)	
NA	8 (44.4%)	10 (55.6%)	
Age, *n* (%)			0.011
≤50	6 (19.4%)	25 (80.6%)	
>50	18 (52.9%)	16 (47.1%)	
NA	3 (50%)	3 (50%)	

FFPE, formalin‐fixed and paraffin‐embedded; CSCC, cervical squamous cell carcinoma; IHC, immunohistochemistry; SCC, squamous cell carcinoma antigen; NA, not available.

Collectively, our results indicated that myCAFs are a crucial component of the TME of CSCC, forming a barrier to protect cancer cells from immune surveillance and clearance. They may also help stimulate cell proliferation and angiogenesis, inhibit apoptosis, and reconstruct the ECM to enhance tumor metastasis (Figure 7C).

## 3 Discussion

Although vaccines and radical hysterectomy are effective measures for preventing and treating cervical cancer, the treatment of recurrent/metastatic cervical cancer remains a major obstacle to achieving the goal of cervical cancer elimination. Herein, we characterized a high‐resolution immunological landscape of CSCC combined with snRNA‐seq and Stereo‐seq technology, which may facilitate the management and treatment of HPV‐induced cervical cancer.

Unraveling the cellular composition of cervical cancer is fundamental for understanding its associated immunological characteristics. However, scRNA‐seq data remain limited for clinical CSCC samples, with public data only available from one patient, as reported by Li et al.^[^ ^31^ ^]^ Moreover, the cell types isolated by Li et al. mainly contained stromal and cancer cells, and <700 immune cells (annotated as macrophages and lymphocytes) were identified. Using snRNA‐seq, we obtained 67 003 cells and identified several cell types, including epithelial cells, stromal cells, and various immune cells present in cervical cancer tissues, which could serve as a valuable resource for research on the cellular diversity of cervical cancers. While we were most interested in the cancerous squamous epithelial cells, the number of CECs (*MUC5B, WFDC2*) was larger than that of the cancer cells (*TP63, KRT5, CDKN2A*) (Figure 1B–D). Anatomical characteristics and sampling bias may have led to the high presentation of CECs. The normal cervix can be divided into the endocervix (adjacent to the uterus) covered by columnar epithelia and the ectocervix (adjacent to the vagina) covered by squamous epithelia. The interface of the two types of epithelia is called the squamocolumnar junction (SCJ), which is the primary region where neoplasia occurs.^[^ ^87^ ^]^ After surgery, the dissected tissues were first provided for clinicopathological diagnosis, and some cancerous tissues, most probably containing the SCJ, were reserved. Samples for this study were obtained later, which might be endocervical tissues above the SCJ that contain mostly columnar epithelia and invasive squamous tumors underneath the mucosa. In addition to the endocervical mucosa, the cervical glands are also composed of CECs. Therefore, sampling site bias and the cellular composition of the endocervix might have contributed to the large number of CECs in our snRNA‐seq data. We also attempted to divide T cells into subpopulations to unravel their detailed functions in CSCC. Unfortunately, because of the low expression percentages of *CD4* and *CD8*, we failed to confidently discriminate them into subpopulations of CD4^+^ and CD8^+^ cells, making it difficult to dissect the detailed interplay between T cell subpopulations and other cells in the CSCC microenvironment. scRNA‐seq or snRNA‐seq with a larger sample size should be used to refine the characterization of subgroups of cell types in CSCC.

Currently, ICB therapies, especially those using PD‐L1/PD‐1 and CTLA4 inhibitors, are among the novel methods for treating metastatic cervical cancers. Several studies have reported wide expression of PD‐L1 in cervical cancers, with positivity rates ranging from 34% to 96%.^[^ ^88^, ^89^, ^90^ ^]^ However, PD‐L1 expression alone was not associated with the disease outcome of patients with cervical cancer.^[^ ^88^ ^]^ Indeed, the response rates to PD‐1/PD‐L1 and CTLA‐4 inhibitors fluctuated greatly among different trials, and the efficacies sometimes seemed independent of the expression status of associated checkpoint genes.^[^ ^14^, ^17^, ^91^ ^]^ In our study, the expression levels of most immune suppressive genes in the tumor and inflammation areas of CSCC were not significantly higher than those in the non‐cancer samples, except for *LGALS9* and *IDO1* (Figure 2D). LGALS9 (i.e., galectin 9) downregulates effector T‐cell immunity by binding to Tim‐3 on the T‐cell surface or by inhibiting the antigen‐presenting ability of DCs.^[^ ^50^, ^51^ ^]^ While disruption of the galectin 9 signaling pathway was shown to induce tumor regression in mice harboring pancreatic ductal adenocarcinoma, a reversed effect was reported in lung metastasis mouse models.^[^ ^92^, ^93^ ^]^ IDO1 is mainly expressed in DCs and helps degrade tryptophan into kynurenine, which suppresses T cell functions.^[^ ^94^ ^]^ It was found that inhibition of IDO1 enhances the radiosensitivity of HeLa and SiHa tumorsphere cells, indicating the potential application of IDO1 inhibitors as radiosensitizers.^[^ ^95^ ^]^ Whether targeting LGALS9 and IDO1 could improve the treatment of CSCC requires further exploration, as other components in the TME, such as myCAFs, may complicate the situation. Our study also revealed suppressive adaptive immunity in tumor areas with low metabolic activity, highlighting the critical role of metabolic modulation in the TME. Recent clinical trials have combined checkpoint inhibitors with metabolic agents that target glucose, amino acids, and nucleotide metabolism.^[^ ^96^ ^]^ A better understanding of the crosstalk between immune responses and metabolism would further benefit cancer therapy.

Owing to tissue heterogeneity, the marker genes for CAFs vary among cancers and CAFs have been classified into diverse subtypes and display distinct functions.^[^ ^55^, ^56^, ^57^, ^58^ ^]^ Previous studies have isolated cancer stromal fibroblasts as a whole or used *α*‐SMA (encoded by *ACTA2*), FAP, and vimentin as staining markers for CAFs in CSCC,^[^ ^59^, ^97^ ^]^ but detailed investigations on the subtypes of CAFs in CSCC remain scarce. This is the first study to describe the spatial‐associated biological properties of myCAFs in clinical samples of CSCC. We showed that *ACTA2*, *POSTN*, *ITGB4*, and *FAP* are adequate marker genes for identifying myCAFs in CSCC (Figure 3B,C), with *POSTN* displaying the highest specificity and prognostic value (Figure S6, Supporting Information). Other genes, such as *NTM, APOLD1, ADAMTS6, ISM1*, and *NKD1* might serve as complementary marker genes for myCAFs in CSCC (Table S7). EMT is the process by which epithelial cells lose their original characteristics and transform into mesenchymal cells, which is a potential origin of CAFs. Some CAFs may induce EMT to enhance the proliferation and invasiveness of cancer cells, as reported in breast cancer.^[^ ^98^ ^]^ The myCAFs from our CSCC samples might be derived from activated normal fibroblasts rather than from EMT (Figure 4A). The myCAF^+^ tumors were active in proliferation, lacked lymphocyte infiltration, and displayed resistance to ICB therapy. Exposing these immune‐evasive tumors to the immune system is essential for the eradication of cancer cells. Researchers have tried to interfere with the activation, action, and normalization processes of CAFs using antibodies or inhibitors in several ongoing clinical trials.^[^ ^99^ ^]^ Since genes highly expressed by CAFs are also essential to normal tissues, their efficacies and side effects require close monitoring. It is interesting to find that not all CSCC samples are positive of myCAFs. Only 28.6% (4/14) of the patients with CSCC in the Stereo‐seq experiment showed the presence of myCAFs, and only 21.1% (15/71) of the FFPE samples in the IHC experiment were positive for POSTN. It might be possible that other subtypes of CAFs in CSCC were not detected due to sampling bias. For example, CAFs with a highly activated metabolic state (meCAFs) are associated with cancer cells using oxidative phosphorylation as a major metabolic route rather than glycolysis and promote metastasis. While the myCAFs characterized in our study suggested resistance to immunotherapy of patients with CSCC, meCAFs predicted a favorable response to immunotherapy of patients with pancreatic ductal adenocarcinoma.^[^ ^55^ ^]^ It would be necessary to dissect the origin, function, and prevalence of other CAF phenotypes in CSCC using more samples, which could help reveal the mechanism of tumor resistance to different therapies.

This study has several limitations. 1) The samples for snRNA‐seq and Stereo‐seq were not paired. We were only able to collect paired samples from one patient with cancer. Individual and anatomical heterogeneity may hinder the comprehensive annotation of cell types. For example, myCAFs were found in four Stereo‐seq samples, but the number of myCAFs was very small in the snRNA‐seq data. 2) Because all cervical cancer cases had progressed to invasive stages, most of the tissues collected mainly contained invasive tumors, making it impossible to compare the intra‐individual differences between normal epithelia and tumors. 3) Unlike tumor cells, the gene expression levels of immunocytes were relatively low, hindering the spatial analysis of most immune cells at high resolution. 4) Our results were drawn from observations of a limited number of clinical samples. Although we incorporated public data and conducted IHC/mIF to confirm the pro‐tumorigenic phenotype of myCAFs, further validation of the functional signaling of myCAFs in CSCC is necessary.

In conclusion, our data demonstrated the high heterogeneity of viral gene expression, immune response, and energy metabolism in CSCC, indicating that combined drugs or therapies targeting multiple biological processes would be a better practice for treating CSCC. Interventions on myCAFs and tumor metabolism may complement the current treatments for CSCC. Further investigations into these biological aspects may facilitate the development of new drugs or therapies for CSCC and other HPV‐induced squamous cell carcinomas.

## 4 Experimental Section

### Patients and Samples

Cervical specimens were collected from 20 patients aged 38–69 years from the Department of Obstetrics and Gynecology of Tongji Hospital in Wuhan and the Department of Obstetrics and Gynecology of Southwest Hospital in Chongqing. Based on colposcopy examination, 18 patients were diagnosed with CSCC (stage IB1 to stage IIIC1), and 2 patients were diagnosed with benign gynecological diseases but also required surgery (Table S1 and Figure S1A, Supporting Information). Carcinoma staging was performed according to the FIGO staging system criteria. Freshly collected samples were used for snRNA‐seq and Stereo‐seq. A total of 71 archived FFPE samples were retrospectively obtained from the Department of Obstetrics and Gynecology of Tongji Hospital in Wuhan to verify the presence of myCAFs in CSCC.

### Single‐Nucleus RNA Sequencing

The collected cervical tissues were quick‐frozen in liquid nitrogen for 30 min and stored in a refrigerator at −80 °C. Nuclei isolation and permeabilization were performed under the guidance of the Chromium Next GEM Single‐Cell Multiome ATAC + Gene Expression User Guide (CG000338). snRNA‐seq libraries were prepared using Chromium Single Cell 3ʹ Reagent Kits v3 (10× Genomics, USA) according to the manufacturer's instructions. Briefly, high‐quality sequencing data were obtained after a series of experimental procedures, including cell counting and quality control, gel beads‐in‐emulsion (GEMs) generation and barcoding, post‐GEM‐RT cleanup, cDNA amplification, gene expression library construction, and NovaSeq platform (Illumina, USA) sequencing.

### Tissue Preparation for Spatial Transcriptomic Experiment

A tissue block with an edge length of < 1 cm was dissected from the surgically removed tissues. The tissue block was then rinsed with cold PBS, immersed in a pre‐cooled tissue storage solution (Miltenyi Biotec, Germany), and embedded with pre‐cooled OCT (Sakura, USA) in a −30 °C microtome (Thermo Fisher, USA) within 30 min after surgery. Three to four serial cryosections of 10 µm thickness were cut from the OCT‐embedded samples for H&E staining, Stereo‐seq library preparation, and IHC staining. Brightfield images of the H&E samples were obtained using a Motic microscope scanner (Motic, China) for histopathological assessment.

### Quality Control of RNA Obtained from OCT‐Embedded Samples

Briefly, 100–200 µm thick sections were cut from each OCT‐embedded sample for total RNA extraction using the RNeasy Mini Kit (Qiagen, USA) according to the manufacturer's protocol. RNA integrity number (RIN) was determined using a 2100 Bioanalyzer (Agilent, USA). Only samples with RIN ≥ 7 were qualified for the transcriptomic study. All samples had an RIN of 7–10.

### Stereo‐seq Library Preparation and Sequencing

The spatial transcriptomic RNA library was constructed using Stereo‐seq capture chips (BGI‐Shenzhen, China) with a size of 1 cm^2^. The capture spots were 220 nm in diameter, with a center‐to‐center distance of 500 nm. Each Stereo‐seq capture probe contained a 25 bp coordinate identity barcode, a 10 bp molecular identity barcode, and a 22 bp polyT tail for in situ mRNA hybridization.^[^ ^38^ ^]^ A cryosection of 10 µm thickness cut from OCT‐embedded tissue was quickly placed on the chip, incubated at 37 °C for 3 min, and then fixed in pre‐cooled methanol at −20 °C for 40 min. The fixed tissue sections were stained with Qubit ssDNA dye (Thermo Fisher, USA) to check the tissue integrity before fluorescent imaging. The tissue sections were then permeabilized using 0.1% pepsin (Sigma, USA) in 0.01 mol/L HCl buffer, incubated at 37 °C for 14 min, and then washed with 0.1× SSC. RNA released from the permeabilized tissue was reverse‐transcribed for 1 h at 42 °C. The tissue sections were then digested with a tissue removal buffer at 42 °C for 30 min. The cDNA‐containing chip was then subjected to cDNA‐release enzyme treatment overnight at 55 °C. The released cDNA was further amplified using a cDNA HIFI PCR mix (MGI). Approximately 20 ng of cDNA was fragmented to 400–600 bp, amplified for 13 cycles, and purified to generate a DNA nanoball library, which was sequenced with the single‐end 50 + 100 bp strategy on an MGI DNBSEQ sequencer (MGI, China).

### IHC Staining

IHC staining of Ki67 and POSTN was performed according to the manufacturer's protocol. The frozen sections were dried at room temperature, placed in an oven at 37 °C for 10–20 min, fixed with 4% paraformaldehyde for 20 min, and washed thrice with PBS (pH = 7.4) for 5 min. The antigens were then repaired with EDTA (pH 9.0) and endogenous peroxidase was blocked with 3% hydrogen peroxide. The slides were blocked with 3% BSA (G5001‐100 g, Servicebio) at room temperature for 30 min and then incubated with Ki67 (ab16667, Abcam, 1:200) or POSTN (ab215199, Abcam, 1:500) at 4 °C overnight. Finally, the frozen slices were subjected to secondary antibody blocking, DAB staining, nuclear restaining, and dehydration. The protein expression levels of Ki67 and POSTN were evaluated under a microscope by professional pathologists. The expression scores of POSTN in the tumor and stromal regions of 71 FFPE samples were measured according to the positivity percentage (0–5% = 0, 6–25% = 1, 26–50% = 2, 51–75% = 3, >75% = 4) and staining intensity (negative = 0, weak = 1, moderate = 2, strong = 3) (Figure 7A,B). The final score was obtained by multiplying the two scores, which ranged from 0 to 12: Negative = 0, weak = 1–4, moderate = 5–8, strong = 9–12 (Table 1).

### mIF Staining

mIF staining was performed to simultaneously detect 1) the location of myCAFs (POSTN), squamous epithelial cells (KRT13), and basal epithelial cells (TP63) and 2) the spatial relationship between the tumor, myCAFs, and immunocytes (CD4^+^ and CD8^+^). Briefly, tissue sections from FFPE samples were dehydrated and antigen repaired, as described for IHC staining. The sections were then incubated with primary antibodies and stained with DAPI (C0060, Solarbio, Beijing). The details of the primary antibodies used are as follows: POSTN (ab215199, Abcam, 1:500), TP63 (ab124762, Abcam, 1:200), KRT13 (ab16112, Abcam, 5 µg/mL), CD4 (RMA‐0620, MXB), and CD8 (RMA‐0514, MXB). Fluorescence images were obtained using a 3D panoramic scanner (DANJIER, HISHTECH Pannoramic 250, Jinan, China) and were visualized using CaseViewer.

### Quality Control and Gene Expression Quantification of snRNA‐seq Data

Raw sequencing files were first processed using CellRanger version v6.0.2 (10× Genomics, USA) to obtain gene expression matrices. After cell calling, droplets containing no cells were excluded based on the number of filtered unique molecular identifiers (UMIs) mapped to each cell barcode. Droplets with low‐quality cells or those with more than one cell were removed. To obtain a gene expression matrix optimized for individual samples, the R package scCancer v2.2.1 was employed to further filter the expression matrix.^[^ ^100^ ^]^ The filtering thresholds were determined by catching outliers from the distribution of four quality spectra, including the number of total UMIs, the number of expressed genes, the percentages of UMIs from mitochondrial genes, and the percentages of UMIs from ribosomal genes. In addition to filtering cells, genes expressed in fewer than three cells were excluded to avoid false‐positive results. The filtering thresholds of the five samples are listed in Table S2, Supporting Information.

### Cell Type Clustering Using Multi‐Sample snRNA‐seq Data

Integrative analysis of the snRNA‐seq data from the five patients was performed using the IntegrateData function in Seurat v4.^[^ ^101^ ^]^ Further analysis, including normalization, log‐transformation, highly variable gene identification, dimension reduction, clustering, and differential expression analysis, were all conducted using default parameters of Seurat except that dims was set at 1:30. Initially, 35 cell clusters were obtained (69,312 cells with 30,996 genes). To ensure reliable identification, cell clusters consisting of fewer than two samples with less than 15 cells per sample were removed. Finally, 14 cell clusters (67,003 cells with 30,996 genes) were identified based on the reported cell‐type marker genes (Table S3, Supporting Information).

### Analysis of Differentially Expressed Genes

The expression of each gene in each cluster was compared against the rest of the clusters using the Wilcoxon rank‐sum test with the FindAllMarkers function in Seurat v4.^[^ ^101^ ^]^ Significantly up‐ or down‐regulated genes were identified using the following criteria: 1) the absolute value of log2 fold change in gene expression level was > 0.25 unless explicitly noted; 2) genes were expressed by > 25% of the cells belonging to the target cluster. 3) The adjusted *p* value was < 0.05.

### Preliminary Processing of Stereo‐seq Data

Stereo‐seq raw data were automatically processed using the BGI Stereomics analytical pipeline (http://stereomap.cngb.org/), in which the reads were decoded, trimmed, deduplicated, and mapped against human and HPV reference genomes. The reference genomes were: Human, GRCh38.p12; HPV16, K02718.1; HPV18, EF202147.1; HPV33, M12732.1; HPV58, D90400.1. Data on the chip area covered by tissue were extracted based on the ssDNA and H&E staining images using the Lasso function of the BGI Stereomics website. It is worth noting that tumor sites usually had much higher overall mRNA levels than the other anatomical areas, leading to a significant imbalance of transcriptomic signals between the tumor areas and the other sites on the Stereo‐seq slides. Therefore, to fully reflect the spatial transcriptomic landscape around the tumor areas, a bin size of 100 (100 × 100 spots, i.e., 49.72 × 49.72 µm) was used as the analytical unit for the annotation of CSCC Stereo‐seq slides, while a bin size of 200 (200 × 200 spots, i.e., 99.72 × 99.72 µm) was used for the non‐CSCC samples. For quality control, the median number of gene types per bin for all chips should be over 1,000. The downloaded data were processed using Seurat v4.^[^ ^101^ ^]^ The criterion of > 200 UMIs per bin was used to remove bins with low expression signals. The data were normalized using the SCTransform function. Dimension reduction was performed using PCA. Unsupervised clustering of bins was performed using UMAP. Sequencing and analytical details are shown in Table S4, Supporting Information.

### Annotation of Bin Clusters in Stereo‐seq Slides

The bin clusters were annotated based on the in situ expression patterns of the marker genes. The spatial expression patterns of genes in Stereo‐seq slides (Figures S1 and S2, Supporting Information) were analyzed using the SpatialFeaturePlot function of Seurat v4.^[^ ^101^ ^]^ The H&E and IHC images were examined by professional pathologists to determine the tissue types. The annotated Stereo‐seq areas were confirmed to be consistent with H&E and IHC assessments and marker gene expression patterns.

### Identification of Viral RNA

Viral reads were mapped against HPV reference genomes using BWA. The genome coverage (covered length/full length of the reference genome) and effective depth (total mapped bases/covered length) of each type were calculated. Only samples with a viral genome coverage > 5% and an effective depth > 50× were deemed HPV‐positive (Tables S2, S4 and Figure S3, Supporting Information).

### Signature Enrichment Analysis of Stereo‐seq Clusters

In the enrichment analysis, the expression scores of signature genes were calculated for individual bins using AddModuleScore (on log‐normalized data) of Seurat v4 with default parameters.^[^ ^101^ ^]^ Pathways and cell types included in the enrichment analysis, with the corresponding reference for gene signatures, were as follows: hypoxia,^[^ ^102^ ^]^ glycolysis,^[^ ^103^ ^]^ lipid metabolism,^[^ ^104^ ^]^ lactic acid metabolism, oxidative phosphorylation (MSigDB, https://www.gsea‐msigdb.org/gsea/msigdb/), pentose phosphate pathway,^[^ ^105^ ^]^ macrophages,^[^ ^106^ ^]^ and other immune cells (Figure 2F,G).^[^ ^107^ ^]^

### Prediction of the Spatial Distribution of Immunocytes

Using the gene signatures of immunocytes as input, the cell type scores of each bin in the tumor areas were calculated using the AddModuleScore of Seurat v4.^[^ ^101^ ^]^ Combining the spatial coordinates of the bins, the possible spatial distribution of the corresponding cell type was obtained (Figure S5D, Supporting Information).

### Signature Enrichment Analysis of Fibroblasts, myCAFs, and Cancer Cells with snRNA‐seq Data

Expression scores of signature genes from MSigDB v7.4 (https://www.gsea‐msigdb.org/gsea/msigdb/) were calculated for individual cells using the AddModuleScore function (on log‐normalized data) of Seurat v4 with default parameters to assess differential pathways in fibroblasts, myCAFs, and cancer cells (Figure 3D).^[^ ^101^ ^]^

### Chromosomal CNV Analysis

InferCNV v1.6.0 (https://github.com/broadinstitute/inferCNV) was used to explore the chromosomal CNV between cancer cells, myCAFs, the other fibroblasts, and CECs using the snRNA‐Seq data (Figure 3F). The expression intensity of genes across chromosomal positions for each cell was compared against the mean value calculated for all cells involved (cancer cells, myCAFs, the other fibroblasts, and CECs). Residual signals were filtered using the default cutoff (0.1), as recommended for scRNA‐seq data.

### Pseudotime Analysis of myCAFs and the Other Fibroblasts

Monocle 2 (version: 2.18.0) was used to explore the development trajectory of myCAFs (234 cells) and the other fibroblasts (9,602 cells). The trajectory was constructed using DEGs with qval < 0.01 (Figure 4A).^[^ ^108^ ^]^ The function differentialGeneTest was then used to extract the genes and performed regression analysis to show their pseudotime expression patterns (Figure 4C). GO enrichment analysis for the modules was conducted using Metascape with the default settings (Figure 4D).^[^ ^109^ ^]^

### Regulatory Network Analysis of myCAFs

GENIE3 (version 1.12.0) was used to explore the gene regulatory network of myCAFs.^[^ ^110^ ^]^ All regulatory factor‐target gene pairs were filtered by weight > 0.05 (Figure 4E). For transcription factors with > 30 targets, GO enrichment analysis of the target genes was performed using Metascape with the default settings (Figure 4F).^[^ ^109^ ^]^

### Multimodal Intersection Analysis (MIA)

To integrate snRNA‐seq and Stereo‐seq data, the overlapping degree of the expression levels of cell type‐specific genes identified by snRNA‐seq data and the area‐specific genes characterized by Stereo‐seq data were measured using the MIA approach (Figure S5A, Supporting Information).^[^ ^111^ ^]^ The lower the *p*‐value, the higher the overlap between a certain cell type and the Stereo‐seq area. MIA was conducted to confirm the consistency between cell types and Stereo‐seq annotated areas and to identify Stereo‐seq areas composed of myCAFs (Figure S5B, Supporting Information).

### DEG and GO Enrichment Analysis of myCAF^+^ and myCAF^–^ Tumor Areas

The expression of each gene in the myCAF^+^ clusters was compared to that in the myCAF^–^ clusters of the same Stereo‐seq chip using the Wilcoxon rank‐sum test with the FindMarkers function of Seurat v4.^[^ ^101^ ^]^ Significantly up‐ or down‐regulated genes (Figure 5B) were identified using the following criteria: 1) the absolute value of log2 fold change in gene expression level was > 0.25 unless explicitly noted; 2) genes were expressed by more than 25% of the bins belonging to the target cluster. 3) The adjusted *p* value was < 0.05. GO enrichment analysis was conducted using Metascape with the default settings (Figure 5D).^[^ ^109^ ^]^

### Cell–Cell Communication between myCAFs and the Other Cell Types in CSCC Tissues

To understand the communication network between myCAFs and other cell types, cell–cell communication was conducted using CellChat with the snRNA‐seq data to obtain the ligand‐receptor pairs regulated by myCAFs (Figure 6A).^[^ ^112^ ^]^ The probability of cell–cell communication was estimated by integrating gene expression with prior knowledge of the interactions between signaling ligands, receptors, and their cofactors.

### Prognostic Analysis of myCAFs with TCGA Data

The gene expression profiles of CSCC were downloaded from TCGA (https://portal.gdc.cancer.gov/) with the latest follow‐up prognostic information obtained from an integrated clinical data resource. Tthe signature score of myCAFs was calculated for each patient with CSCC with GSVA using the marker genes of myCAFs (*ACTA2, POSTN, ITGB4*, and *FAP*). Based on the median GSVA score, patients were divided into two groups: high myCAF signatures versus low myCAF signatures. Kaplan–Meier overall survival and progression‐free survival curves were generated using GraphPad Prism 6 (Figure 6D,E). Correlation analysis between *POSTN/ITGB4* expression and survival probabilities were carried out similarly (Figure S6, Supporting Information).

### Estimating the Effects of myCAFs on Immunotherapy in CSCC

To explore the impact of myCAFs on immunotherapy in CSCC, the RNA‐seq data of 252 patients with CSCC were downloaded from TCGA. The Maftools package was used to generate the TMB score of each patient.^[^ ^113^ ^]^ The MSI scores were determined by Li et al. with MSIsensor.^[^ ^114^, ^115^ ^]^ ImmuCellAI was used to predict the response rate of ICB therapy based on the gene expression matrix of the 252 patients.^[^ ^116^ ^]^ The relationships between *POSTN* expression and TMB score, MSI score, or immune gene expression were estimated by Spearman's correlation coefficients (Figure 6F–H).

### Statistical Analysis

Comparisons of the GSVA scores between the myCAF^+^ tumor and myCAF^–^ tumor, and between hypermetabolic tumor and hypometabolic tumor were conducted using Student's *t* test and Kruskal–Wallis test in R 4.1.0, respectively. Other tests, including Wilcoxon's rank‐sum test, Wilcoxon signed‐rank test, and Chi‐square test, were all conducted in R 4.1.0. *p* < 0.05 was considered statistically significant. Asterisks indicate the significance levels of *p* values: ns, not significant; *p < 0.05; **p < 0.01; ***p < 0.001; ****p < 0.0001.

### Ethical Statement

This study was reviewed and approved by the Medical Ethics Committee of Tongji Medical College, Huazhong University of Science and Technology (TJ‐IRB20210609), Southwest Hospital, Third Military Medical University (KY2020142), and the Institutional Review Board of Beijing Genomics Institute, Shenzhen, China (BGI‐IRB 21050).

## Conflict of Interest

A. Chen and M. Cheng are applying for patents covering the chip, procedure, and applications of Stereo‐seq. The other authors declare that they have no competing interests.

## Author Contributions

Z.O., S.L., J.Q., W.D., P.R., D.C., and J.W. contributed equally to this work. Z.O., P.W., J.L., W.D., and D.C. designed this study. P.W., Z.O., J.L., X.X., D.M., X.J., H.Y., and J.W. supervised this study. P.W., Y.W., W.D., and S.L. coordinated the sample collection. S.L., Y.D., and T.P. conducted snRNA‐seq and IHC experiments. P.R., Y.T., and D.W. performed Stereo‐seq library construction and H&E staining. A.C. and M.C. provided technical support for Stereo‐seq experiments. H.L. conducted sequencing for Stereo‐seq libraries. J.Q., J.W., Y.T., and D.W. conducted the data analysis. Z.O., S.L., P.R., J.Q., and J.W. wrote the original manuscript. Z.O., S.L., P.W., J.L., W.D., and D.C. reviewed and polished the manuscript.

## Supporting information

Supporting Information

Supplemental Table

## Data Availability

The data and scripts supporting the findings of this study have been deposited into CNSA (CNGB Sequence Archive) of CNGBdb (https://db.cngb.org/cnsa/): Stereo‐seq data, CNP0002543; snRNA‐seq data, CNP0002535. The dataset used to verify the distinguishing ability of the DEGs between preinvasive and invasive cancerous lesions was obtained from the Gene Expression Omnibus (GEO) database (www.ncbi.nlm.nih.gov/geo) by the Accession Number of GSE63514.
