# Supplementary material for: Single‐Nucleus RNA Sequencing and Spatial Transcriptomics Reveal the Immunological Microenvironment of Cervical Squamous Cell Carcinoma
Source: Adv Sci (Weinh). 2022 Aug 19;9(29):2203040. doi: 10.1002/advs.202203040 (PMC9561780; doi:10.1002/advs.202203040)
Supplement: Supplementary file 1 — Supporting Information [file ADVS-9-2203040-s002.pdf]

ADVANCED SCIENCE
Open Access

# Supporting Information

for *Adv. Sci.*, DOI 10.1002/advs.202203040

Single-Nucleus RNA Sequencing and Spatial Transcriptomics Reveal the Immunological Microenvironment of Cervical Squamous Cell Carcinoma

*Zhihua Ou, Shitong Lin, Jiaying Qiu, Wencheng Ding, Peidi Ren, Dongsheng Chen, Jiaxuan Wang, Yihan Tong, Di Wu, Ao Chen, Yuan Deng, Mengnan Cheng, Ting Peng, Haorong Lu, Huanming Yang, Jian Wang, Xin Jin, Ding Ma, Xun Xu*, Yanzhou Wang*, Junhua Li* and Peng Wu**

Supporting Information

**Single-nucleus RNA Sequencing and Spatial Transcriptomics Reveal the Immunological Microenvironment of Cervical Squamous Cell Carcinoma**

*Zhihua Ou, Shitong Lin, Jiaying Qiu, Wencheng Ding, Peidi Ren, Dongsheng Chen, Jiaxuan Wang, Yihan Tong, Di Wu, Ao Chen, Yuan Deng, Mengnan Cheng, Ting Peng, Haorong Lu, Huanming Yang, Jian Wang, Xin Jin, Ding Ma, Xun Xu,[*] Yanzhou Wang,[*] Junhua Li,[*] Peng Wu[*#]*

Z. Ou, S. Lin, J. Qiu, W. Ding, P. Ren, D. Chen, and J. Wang contributed equally to this work.

[#]Lead contact

[*]Correspondence: pengwu8626@tjh.tjmu.edu.cn (P.Wu), lijunhua@genomics.cn (J.Li), w.y.z@foxmail.com (Y.Wang), xuxun@genomics.cn (X.Xu)

Z. Ou, J. Qiu, P. Ren, D. Chen, J. Wang, Y. Tong, D. Wu, A. Chen, M. Cheng, H. Lu, H. Yang, J. Wang, X. Jin, X. Xu, J. Li
BGI-Shenzhen, Shenzhen 518083, China.
Email: lijunhua@genomics.cn; xuxun@genomics.cn

S. Lin, W. Ding, T. Peng, D. Ma, P. Wu
Cancer Biology Research Center (Key Laboratory of the Ministry of Education), Tongji Hospital, Tongji Medical College, Huazhong University of Science and Technology, Wuhan 430000, China.
Department of Gynecologic Oncology, Tongji Hospital, Tongji Medical College, Huazhong University of Science and Technology, Wuhan 430000, China.
Email: pengwu8626@tjh.tjmu.edu.cn

Y. Deng, Y. Wang
Department of Obstetrics and Gynecology, Southwest Hospital, Third Military Medical University, Chongqing 400038, China.
Email: w.y.z@foxmail.com

Z. Ou, P. Ren, J. Wang, Y. Tong, D. Wu, J. Li

Shenzhen Key Laboratory of Unknown Pathogen Identification, BGI-Shenzhen, Shenzhen 518083, China.
Email: lijunhua@genomics.cn

J. Qiu, M. Cheng
College of Life Sciences, University of Chinese Academy of Sciences, Beijing 100049, China.

Y. Tong
College of Innovation and Experiment, Northwest A&F University, Yangling 712100, China.

D. Wu
School of Basic Medicine, Qingdao University, Qingdao 266071, China.

A. Chen
Department of Biology, University of Copenhagen, Copenhagen DK-2200, Denmark.

H. Lu
China National GeneBank, BGI-Shenzhen, Shenzhen 518120, China.

H. Yang, J. Wang
James D. Watson Institute of Genome Sciences, Hangzhou 310058, China.

H. Lu, X. Xu
Guangdong Provincial Key Laboratory of Genome Read and Write, Shenzhen 518120, China.
Email: xuxun@genomics.cn

**1. Supporting Tables (see Excel files)**

Table S1. Clinical characteristics and experimental details for samples.

Table S2. snRNA-seq data statistics.

Table S3. Marker genes for the annotation of cell types in CSCC.

Table S4. Stereo-seq data statistics.

Table S5. Expression matrix of immune genes in Stereo-seq areas.

Table S6. GSVA gene sets used to characterize the metabolic statuses of the ST tumor areas.

Table S7. DEGs for fibroblasts, myCAFs, and cancer cells.

Table S8. DEGs between myCAFs and the other fibroblasts.

Table S9. Regulatory genes and their targets identified in myCAFs (weight > 0.05).

Table S10. DEGs for myCAF+ and myCAF- tumors.

## 2. Supporting Figures

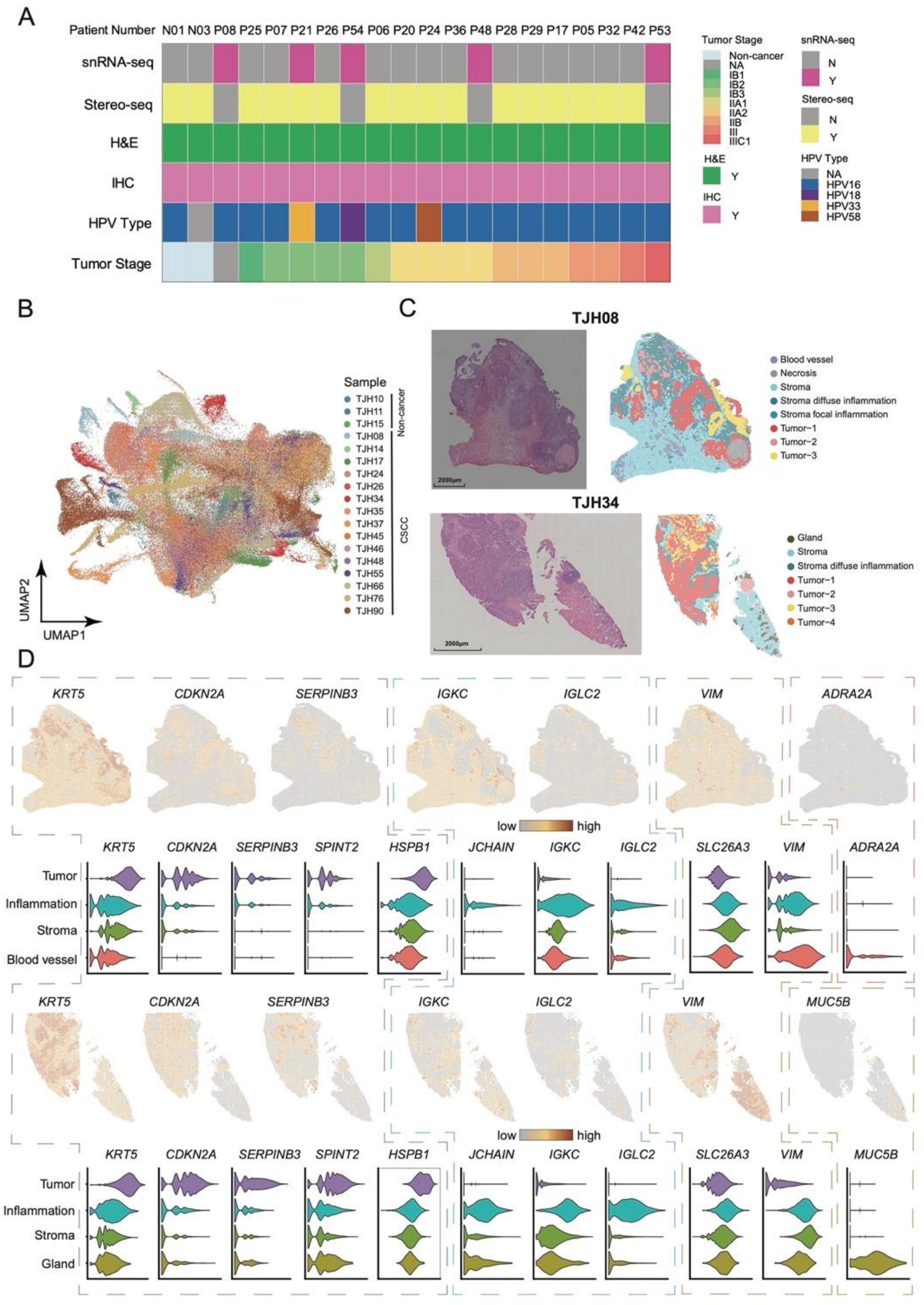

**Figure S1. Experimental details and the annotation process of Stereo-seq slides.** (A) Clinical characteristics and experimental details of cervical samples from CSCC and non-cancer patients. (B) UMAP of Stereo-seq bins from 18 cervical samples. (C) Annotation results of two representative Stereo-seq slides. (D) Expression of tissue-specific genes in two representative Stereo-seq slides. The outline color indicates different tissue types: purple, tumor; blue, stroma with inflammation; green, stroma; red, blood vessel; brown, gland. Violin plots display the gene expression levels in the Stereo-seq areas.

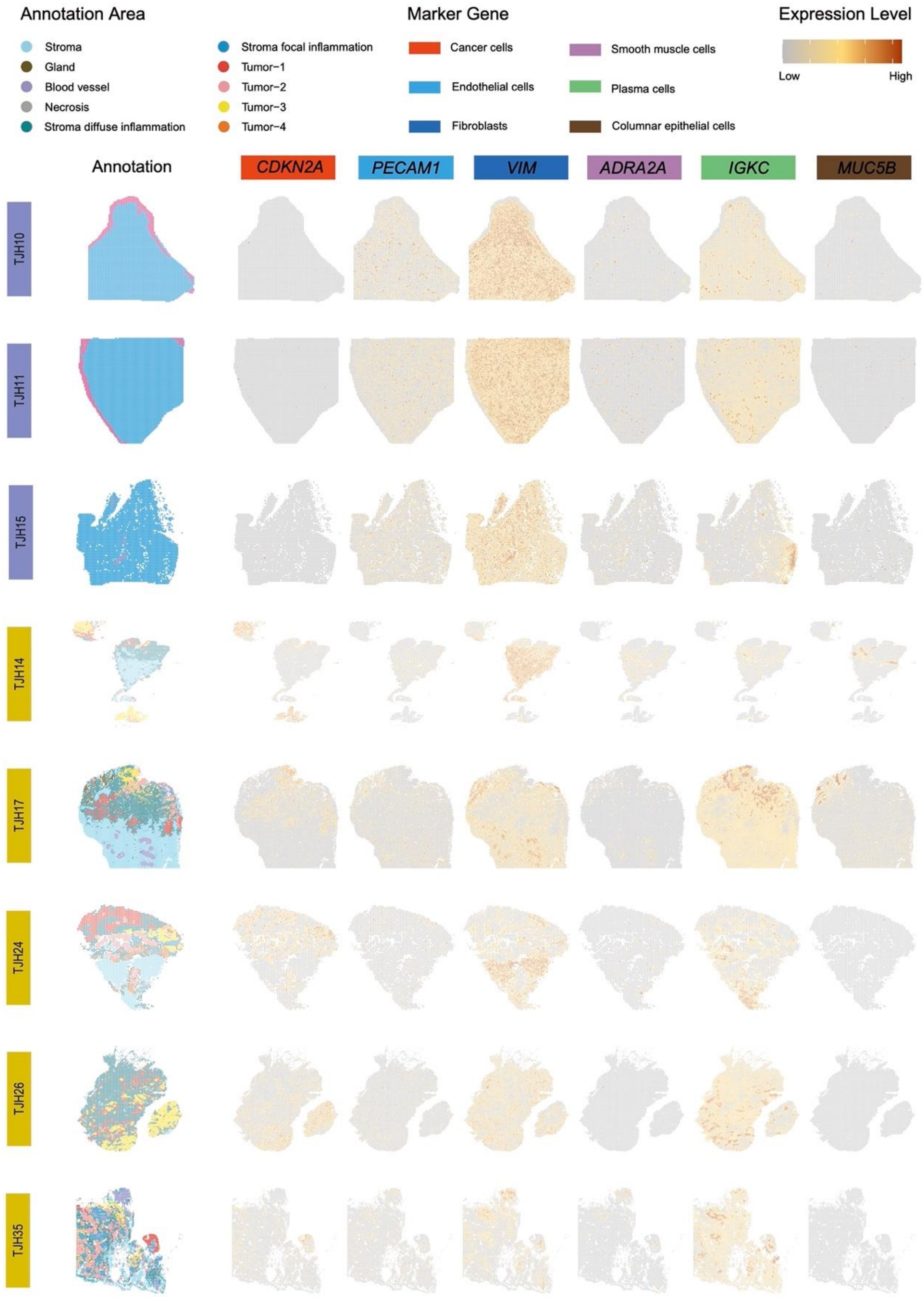


**Figure S2 (To be continued). Annotation results of the 18 Stereo-seq slides.** Non-cancer samples included TJH10, TJH11, and TJH15. All the other samples were CSCC samples. Six genes were selected to represent different tissue types. Cancer cells, *CDKN2A*; Endothelial cells, *PECAM1*; Fibroblasts, *VIM*; Smooth muscle cells, *ADRA2A*; Plasma cells, *IGKC*; Columnar epithelial cells, *MUC5B*.

**Figure S2 (Continued)**

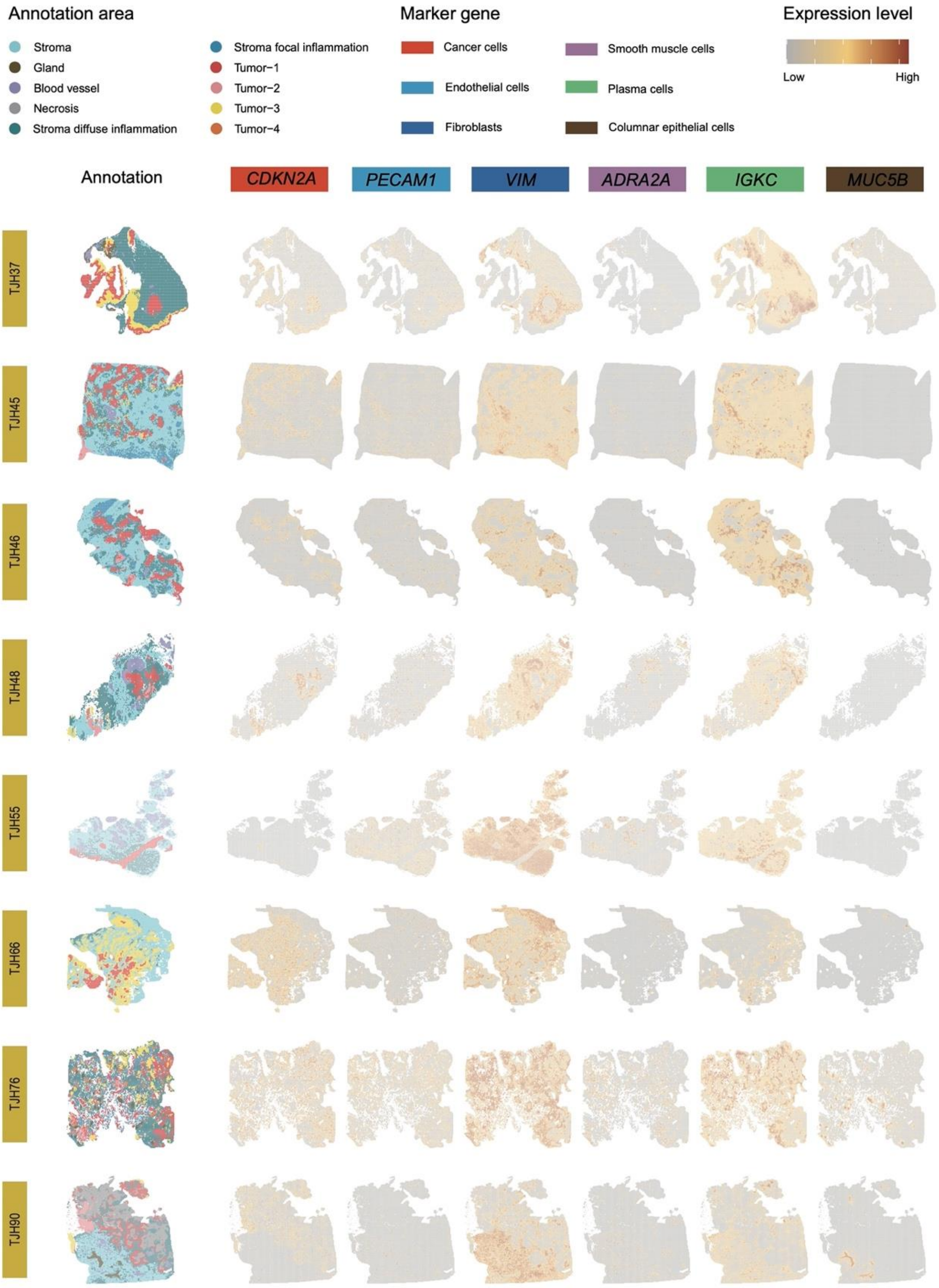

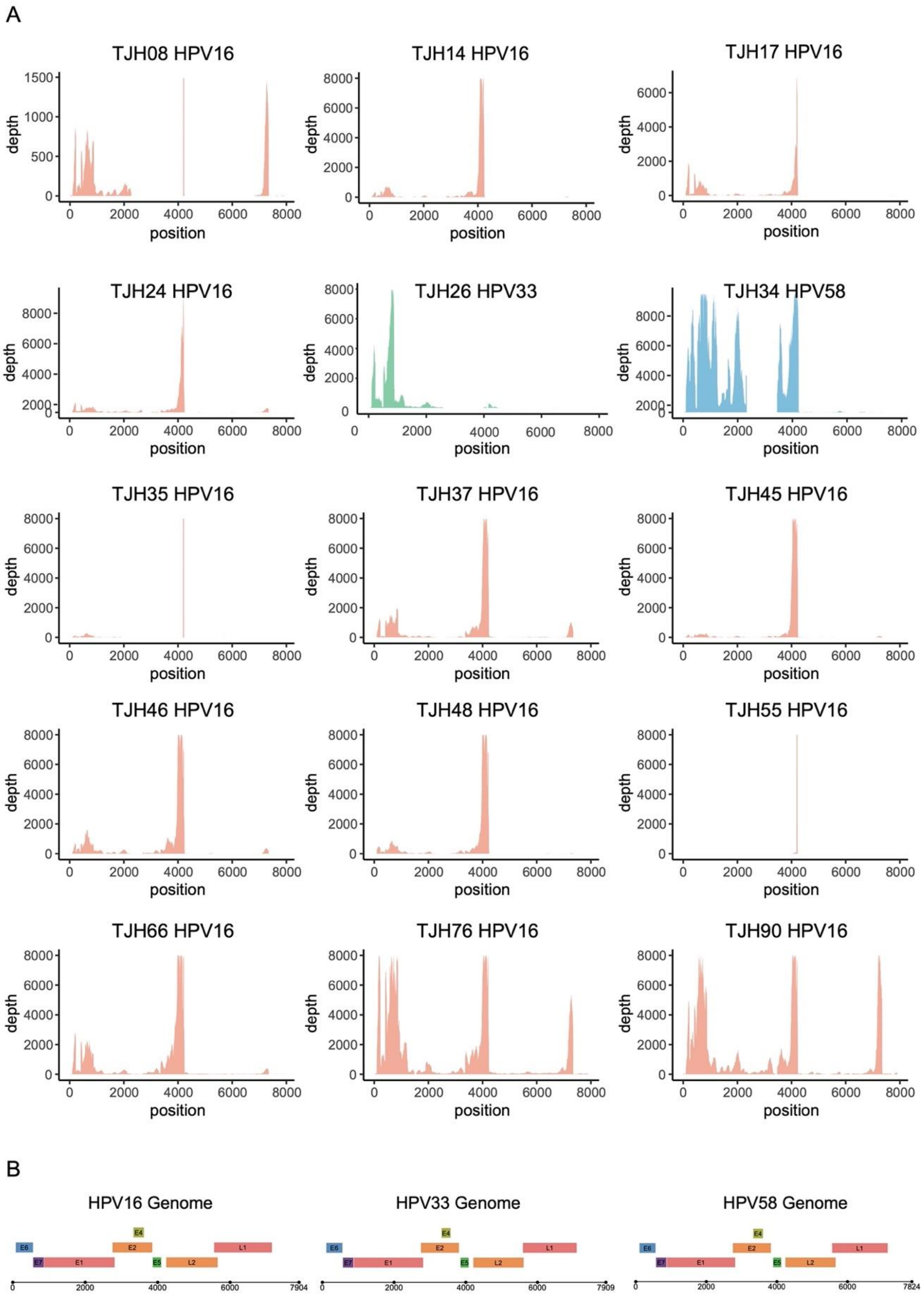


**Figure S3. HPV reads in the Stereo-seq sequencing data of 15 CSCC tissues.** (A) Mapping of HPV reads against the corresponding reference genome. (B) Schematic plot showing the genomic arrangement of the HPV genes in a linear form.

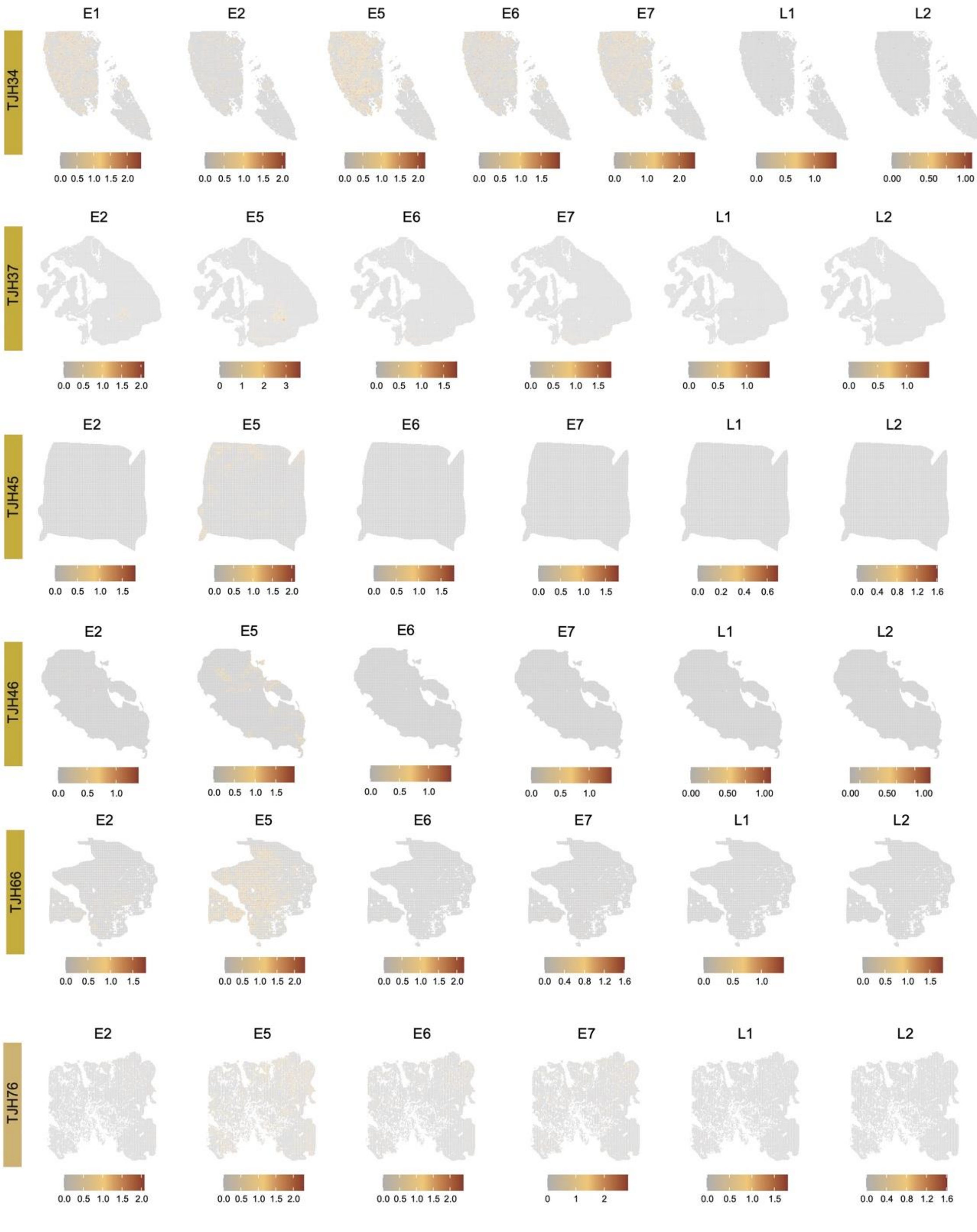


**Figure S4. Expression of HPV genes in the tumor areas of selected CSCC Stereo-seq slides.** The annotation result for each sample can be found in **Figure S2**.

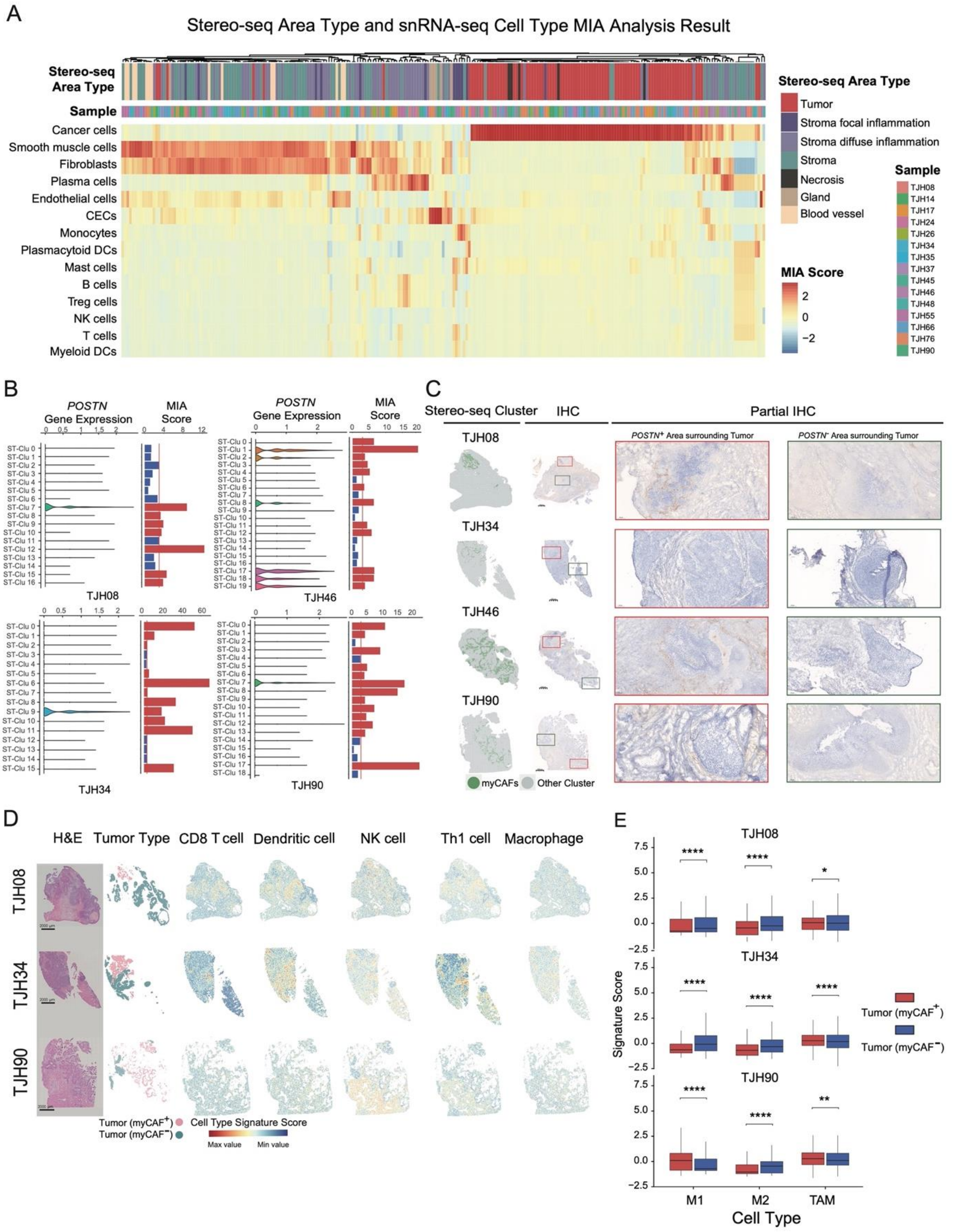


**Figure S5. Characterization of myCAFs**. (A) Correlation between snRNA-seq cell types and the Stereo-seq areas defined by multimodal intersection analysis (MIA). (B) Expression of *POSTN* in Stereo-seq clusters and the associated MIA scores for myCAFs. (C) Spatial clustering of myCAFs in Stereo-seq slides and the IHC staining of POSTN in corresponding serial sections of CSCC samples. (D) Spatial prediction of immunocytes in Stereo-seq slides.

(E) The abundance of macrophages including M1 (tumor-suppressive phenotype macrophage), (tumor-promoting phenotype macrophage) M2, and TAM (tumor-associated macrophage) in myCAF$^+$ and myCAF$^-$ tumors. TJH08, 5428 bins (472 myCAF$^+$ tumor bins and 4956 myCAF$^-$ tumor bins); TJH34, 4617 bins (1905 myCAF$^+$ tumor bins and 2712 myCAF$^-$ tumor bins); TJH90, 3863 bins (2729 myCAF$^+$ tumor bins and 1134 myCAF$^-$ tumor bins). The $p$ values were determined by Student's $t$ test: ns, not significant; *$p < 0.05$; **$p < 0.01$; ***$p < 0.001$; ****$p < 0.0001$.

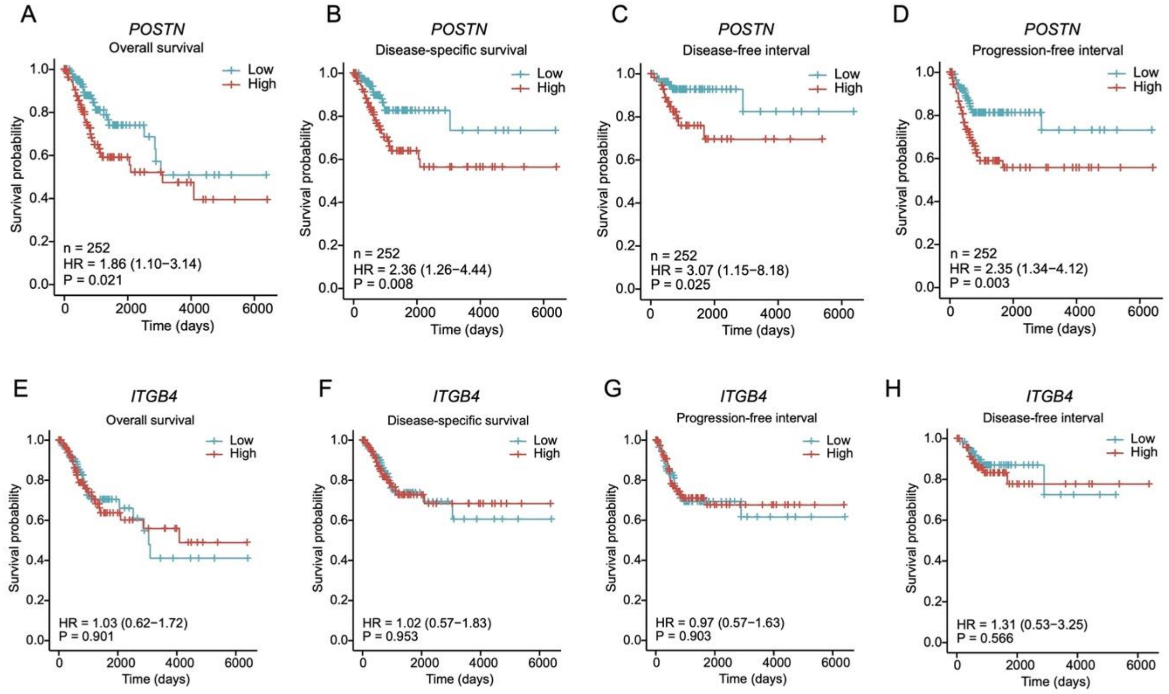


**Figure S6. Correlation analysis between *POSTN/ITGB4* expression level and survival probabilities of patients with CSCC.** The analysis was based on a TCGA dataset containing 252 patients with CSCC. (A-D) The expression level of *POSTN* was negatively associated with the overall survival probability, disease-specific survival probability, disease-free interval survival probability, and progression-free interval survival probability of patients with CSCC. (E-H) No significant correlation was found between the expression level of *ITGB4* and the overall survival probability, disease-specific survival probability, disease-free interval survival probability, or progression-free interval survival probability of patients with CSCC.
